# Single-Cell and CellChat Resolution Identifies Collecting Duct Cell Subsets and Their Communications with Adjacent Cells in PKD Kidneys

**DOI:** 10.3390/cells12010045

**Published:** 2022-12-22

**Authors:** Linda Xiaoyan Li, Xu Zhang, Hongbing Zhang, Ewud Agborbesong, Julie Xia Zhou, James P. Calvet, Xiaogang Li

**Affiliations:** 1 Department of Internal Medicine, Mayo Clinic, Rochester, MN 55905, USA; 2 Department of Biochemistry and Molecular Biology, Mayo Clinic, Rochester, MN 55905, USA; 3 Robert and Arlene Kogod Center on Aging, Mayo Clinic, Rochester, MN 55905, USA; 4 Department of Physical Medicine & Rehabilitation, Mayo Clinic, Rochester, MN 55905, USA; 5 Department of Biochemistry and Molecular Biology, University of Kansas Medical Center, Kansas City, KS 66160, USA

**Keywords:** ADPKD, single cell sequencing, *Pkd1* mutation, CellChat

## Abstract

ADPKD is a genetic disorder with a molecular complexity that remains poorly understood. In this study, we sampled renal cells to construct a comprehensive and spatiotemporally resolved gene expression atlas in whole *Pkd1* mutant polycystic mouse kidneys at single-cell resolution. We characterized cell diversity and identified novel collecting duct (CD) cell subtypes in cystic kidneys. We further found that CD cells appear to take different cell fate trajectories, and the first and the most important step might take place around day 14 in *Pkd1* homozygous kidneys. After that day, increased numbers of CD cells showed highly proliferative and fibrotic characteristics, as detected in later-stage *Pkd1* homozygous kidneys, both of which should contribute to cyst growth and renal fibrosis. With a newly developed modeling algorithm, called CellChat Explorer, we identify cell-to-cell communication networks mediated by the ligand receptor, such as MIF-CD44/CD74, in cystic kidneys, and confirm them via the expression patterns of ligands and receptors in four major cell types, which addresses the key question as to whether and how *Pkd1* mutant renal epithelial cells affect their neighboring cells. The allele-specific gene expression profiles show that the secretion of cytokines by *Pkd1* mutant epithelial cells may affect the gene expression profiles in recipient cells via epigenetic mechanisms, and *vice versa*. This study can be used to drive precision therapeutic targeting of ADPKD.

## 1. Introduction

Autosomal dominant polycystic kidney disease (ADPKD) is the most common cause of inherited kidney failure, affecting >1:1000 individuals worldwide [1,2]. ADPKD results from mutations in one of two genes, PKD1 and PKD2, which encode polycystin-1 (PC1) and polycystin-2 (PC2), respectively [3,4]. The sustained proliferation of inappropriately differentiated PKD mutant renal tubular epithelial cells together with increased cyst-filling fluid secretion contribute to cyst formation and progression in ADPKD, and to the compression and destruction of surrounding normal tubules. Among those ADPKD patients, half of them reach end-stage renal disease by the sixth decade of life [5]. The cellular events and signaling pathways associated with PKD gene mutation include calcium signaling [6,7,8], cyclic AMP (cAMP) [7], c-Myc [9], TWEAK [10], Wnt/β-catenin [11], Id2/Rb [12], mTOR [13], JAK-STATs [14,15], epidermal growth factor (EGF) receptor [16], RNA helicase [17], NF-κB [18], cytokines [19,20], and epigenetic regulators, such as HDAC6, Sirt1 and Smyd2 [16,21,22]. However, it remains to be addressed how and when these pathways interact during cyst growth and progression, and in what renal cell types.

Unlike in the human disease, *Pkd1* mutations in the mouse are recessive [23], thus offering the opportunity to investigate the effects of *Pkd1* mutations in both heterozygotes and homozygotes as well as wildtype mice. Investigating how a cystic cell that harbors *Pkd1* mutations differs from non-cystic heterozygous cells and wildtype cells should facilitate our understanding of disease mechanisms and help spur the development of therapeutic strategies for ADPKD. Unfortunately, since PKD kidneys are a complex mixture of cystic and non-cystic cells, the detection and manipulation of individual cystic cells from renal tissues in PKD animal models has not been feasible. Now, though, recent breakthroughs in single-cell RNA-seq (scRNA-seq) have made it possible to monitor gene expression changes in single cells, paving the way for exploring cellular heterogeneity in PKD kidneys. scRNA-seq offers multiple advantages over previous approaches to studying gene expression in PKD kidneys, including that 1) it is inherently unbiased with respect to the identity of the specific cell-types examined; 2) it generates data with remarkable specificity and statistical power, allowing the definition of individual renal cells; 3) it generates simultaneous measurements across the spectrum of cell types in PKD kidneys; and 4) it provides insights as to whether an individual PKD mutant cell affects the functions of adjacent renal cells in the context of the cystic microenvironment. As such, scRNA-seq analysis allows the discovery of both cell-autonomous and cell-non-autonomous changes in gene expression in different renal cell types in PKD kidneys.

With the goal of characterizing the complex cellular heterogeneity and systemically analyzing gene expression profiles of each cell type in *Pkd1* heterozygous (HET) and *Pkd1* homozygous (HOMO) kidneys, here, we profile 64,280 single-cell transcriptomes across different stages of *Pkd1* HET and HOMO kidneys. By comparing gene expression profiles between age-matched *Pkd1* HET and HOMO kidneys in parallel, and across different stages of *Pkd1* HET and *Pkd1* HOMO kidneys, we have been able to identify different cell populations and different collecting duct cellular subtypes in *Pkd1* HET and HOMO kidneys. We have also identified genes associated with signaling pathways related to cyst initiation and progression, and have addressed a potential mechanism for how the deletion of *Pkd1* in collecting duct cells might affect gene expression in neighboring cells, including in macrophages, fibroblasts, natural killer (NK) and T cells. This study gives a single-cell view of the *Pkd1*-associated PKD pathology, thus providing a unique cellular-level view of transcriptional alterations associated with *Pkd1* heterozygous kidney development and *Pkd1* homozygous cyst initiation and progression, which reveals cell-type-specific and shared gene expression perturbations, disease-associated cellular subpopulations and transcriptional responses to cystic microenvironment. Additionally, this single-cell RNA-seq approach has detected unique cell states within cystic kidneys, and has redefined cellular heterogeneity in renal collecting ducts.

## 2. Material and Methods

### 2.1. Mice

Animal studies were approved by the Institutional Animal Care and Use Committee (IACUC) of Mayo Clinic. *Pkd1^fl/fl^:Pkhd1-Cre* mice were used as HOMO animals and *Pkd1^fl/+^:Pkhd1-Cre* mice were used as HET animals in this study. *Pkd1^fl/fl^:Pkhd1-Cre* mice were generated by cross-breeding *Pkd1^fl/+^:Pkhd1-Cre* female mice with *Pkd1^fl/+^:Pkhd1-Cre* male mice [24]. Genotyping was confirmed by toe PCR using published primers [25].

### 2.2. Antibodies and Immunofluorescence Staining

Paraffin-embedded sections (5 μm) were subjected to staining. For Col1α1 staining, a polyclonal rabbit anti-COL1A1 antibody (GTX112731, GeneTex; 1:100 dilution), a AF555 conjugated secondary antibody (A-31572, Thermo Fisher; 1:500 dilution), and DAPI-contained mounting regent were used. Anti-CENPF (NB500-101, Novus Biologicals, CO, USA), anti-fibronectin (sc-8422, SantaCruz, TX, USA.) and corresponding secondary antibodies were used according to the manufacture’s instruction. Citrate Buffer (pH = 6.0) was used for antigen retrieval, tris-buffered saline (TBS, pH = 7.5) was used as wash buffer, and corresponding isotype control antibodies were used as negative control. Images were analyzed with a NIKON Ti2E microscope.

### 2.3. Preparation of Single-Cell Suspension

Kidneys were dissected, minced into approximately 1 mm^3^ cubes and digested using the Multi Tissue dissociation kit (Miltenyi, 130-110-201). Up to 0.25 g of the tissue was digested with 50 μL of Enzyme D, 25 μL of Enzyme R and 6.75 μL of Enzyme A in 1 mL of RPMI and incubated for 30 min at 37 degrees Celsius. The reaction was deactivated by adding 10% FBS for 3–5 min at 37 degrees Celsius. The solution was then passed through a 40 μm cell strainer (Falcon, 352340). After centrifugation at 1000 RPM for 5 mins, the cell pellet was incubated with 1 mL of RBC lysis buffer (BD, 555899) on ice for 3 mins. The cells were spun down at 1000 rpm for 5 min [26]. The supernatant was removed completely, and the cell pellet was resuspended in 100 μL of Dead Cell Removal MicroBeads (Miltenyi, 130-090-101) per approximately 10^7^ total cells. It was mixed well and incubated for 15 min at room temperature. The binding buffer and MACS^®^ Columns in the dead cell removal kit (Miltenyi, 130-090-101) were prepared during the incubation time. The 20× Binding Buffer Stock Solution was diluted with sterile, double distilled water. Usually, 10 mL 1× binding buffer is necessary for each sample. For cell isolation, we chose a positive selection column type LS (for up to 10^8^ dead cells and up to 2 × 10^9^ total cells) and placed the column in the magnetic field of a suitable MACS^®^ Separator. We prepared the column by rinsing with 1× Binding Buffer (LS: 3 mL), and applied the cell suspension in a suitable amount of 1× Binding Buffer onto the column (LS: 2–3 mL). We let the negative cells pass through. We rinsed with an appropriate amount of 1× Binding Buffer (LS: 3–5 mL), collected effluent as the live cell fraction, spun down the live cells at 1000 rpm for 5 min, and resuspended the cells with a suitable volume of PBS buffer (containing 0.04% BSA). We then applied these cells for cell viability detection and subsequent library preparation once we passed the threshold of cell viability. This method generated single-cell suspensions with greater than 85% viability.

### 2.4. Single-Cell RNA Sequencing: Barcoding and cDNA Synthesis

The single-cell suspension was loaded onto a well on a 10× Chromium Single-Cell instrument (10× Genomics). Barcoding and cDNA synthesis were performed according to the manufacturer’s instructions by the medical genome facility at the Mayo Clinic. Briefly, the 10×™ GemCode™ Technology partitions thousands of cells into nanoliter-scale Gel Bead-In-Emulsions (GEMs), where all the cDNA generated from an individual cell shares a common 10× barcode. In order to identify the PCR duplicates, a Unique Molecular Identifier (UMI) was also added. The GEMs were incubated with enzymes to produce full-length cDNA, which was then amplified by PCR to generate enough for library construction.

### 2.5. Single-Cell RNA Sequencing: Library Construction and Quality Control

The cDNA libraries were constructed using the 3′ version 3 single-cell kit (PN-1000268, 10× Genomics) according to the manufacturer’s original protocol. The final single-cell 3′ library contains standard Illumina paired-end constructs (P5 and P7), a Read1 (R1) primer sequence, a 16 bp 10× barcode, a 10 bp randomer, 100 bp cDNA fragments, an R2 primer sequence and an 8 bp sample index. For post-library construction QC, 1 μL of sample was diluted 1:10 and run on the Agilent Bioanalyzer High-Sensitivity chip for qualitative analysis. For quantification, the Illumina Library Quantification Kit (KAPA Biosystems, Cat# KK4824) was used.

### 2.6. Single-Cell RNA Sequencing and Generation of Data Matrix

The libraries were sequenced on Illumina HiSeq 4000 paired-end kits using the following read length: 26 bp Read1 for cell barcode and UMI, 8 bp I7 index for sample index and 100 bp Read2 for transcript. Cell Ranger 1.3 (http://10xgenomics.com, accessed on 30 June 2020) was used to process the Chromium single-cell 3′ RNA-seq output. First, “cellranger mkfastq” demultiplexed the sequencing samples based on the 8 bp sample index read to generate fastq files for Read1 and Read2, followed by the extraction of a 16 bp cell barcode and a 10 bp UMI. Second, “cellranger count” aligned Read2 to the mouse reference genome (mm10) using STAR [27].

### 2.7. Data Quality Control and Preprocessing

Once the gene–cell data matrix was generated, poor-quality cells were excluded, such as cells with <100 or >5000 unique expressed genes (as they are potentially cell duplets), and SoupX was performed to remove ambient RNA [28], while DoubletFinder was used to detect and remove doublet cells to improve differential gene expression analysis performance [29]. After stringent quality control analyses to remove unwanted cells that were empty droplets or debris, and patch correction, we sequenced a total of 64,280 cells from whole *Pkd1* HET and *Pkd1* HOMO kidneys. Only genes expressed in 10 or more cells were used for further analysis. Cells were also discarded if their mitochondrial gene percentages were over 50%, resulting in 21,701 genes across 45,527 cells. The data were natural log-transformed and normalized for scaling the sequencing depth to a total of 1 × 10^4^ molecules per cell, followed by regressing-out the number of UMI using the Seurat package version 3.6.2 (Developed by Paul Hoffman, Satija Lab and Collaborators. New York Genome Center, NY, USA).

### 2.8. Dimensionality Reduction and tSNE and UMAP Visualization

The Seurat R package (version 3.6.2) was used for dimensionality reduction analysis. We first identified highly variable genes across the single cells, after controlling for the relationship between average expression and dispersion. Fifty statistically significant PCs were selected as input for t-Distributed Stochastic Neighbor Embedding (tSNE) and uniform manifold approximation and projection (UMAP) [30,31]. tSNE visualized the single cells on a two-dimensional space based on expression signatures of the variable genes, and therefore similar to PC loadings. Both clustering methods identified similar cell clusters, expressing the same groups of marker genes with limited variations in cell separation, whereas UMAP places related cell types (clusters) near one another.

### 2.9. Cell Clustering Analysis

The density-based spatial clustering algorithm, DBSCAN [32], was used to identify cell types on the tSNE map with the initial setting for the eps value at 0.5. Clusters were removed if the number of cells was less than 10 and if the cluster was not composed of more than 6 batches. These pruning steps ensured that the resulting clusters were not derived from over-clustering or potential artifacts such as batch effects. When the final 19 clusters were determined, every pair had more than 10 differentially expressed genes (average expression difference > 1 natural log with an FDR corrected *p* < 0.01). Clusters were merged if any pairs had less than 10 differentially expressed genes (average expression difference > 1 natural log with an FDR corrected *p* < 0.01).

### 2.10. Identification of Differentially Expressed Genes and Marker Genes

Cell-specific marker genes were identified in two stages. The first sets of differentially expressed genes (DEGs) were identified by comparing cells in a specific cell type with cells in all other cell types (Seurat package likelihood ratio test: average expression difference > 0.5 natural log with an FDR corrected *p* < 0.01). Next, cells in a specific cell type from one sample were compared to cells in this specific cell type from every other sample in a pairwise manner to identify a second set of DEGs (Seurat package likelihood ratio test: average expression difference > 0.25 natural log with *p* < 0.05). Cell-specific markers were identified by overlapping the first and second sets of DEGs. Since different cells in the kidney share some well-known markers (transitional cells vs. intercalated cells and proximal tubule vs. novel cells), the combination of these two approaches using the lower threshold enabled us to retain the shared markers while identifying distinct markers compared to other cells.

### 2.11. Comparison of the Single-Cell with Bulk RNA Sequence Data

For bulk RNA sequencing analysis, we set the cluster ID as the sample ID for each sample from single-cell RNA sequencing data, and the comparisons between samples were counted as bulk RNA comparisons. The first sets of DEGs (identified as described above) were used for correlation analysis. Z-scores were calculated for normalized expression values for each gene in the single-cell data and for FPKM values of each gene in the bulk RNA sequencing data.

### 2.12. Single Cell Trajectory Analysis

To construct single-cell pseudotime trajectories and to identify gene expression changes as the cells undergo transition, the Monocle 2 (version 2.18.0. Developed by Cole Trapnell lab. Seattle, WA, USA) algorithm was applied to the cell subtypes of the collecting duct cells extracted from kidneys on days 7, 14 and 21 [33]. Monocle introduces a strategy called single-cell trajectory analysis, which uses an algorithm to learn the sequence of gene expression changes that each cell must go through as part of a dynamic biological process to enable us to see these states in each cell. An overall “trajectory” of gene expression changes can place each cell at its proper position in the trajectory analysis [33]. Genes for ordering cells were selected if they were expressed in ≥ 10 cells, their mean expression value was ≥0.05, and their dispersion empirical value was ≥2. Cells were ordered along the trajectory and their trajectory was visualized in the reduced dimensional space. Significantly changed genes along the pseudotime trajectory were identified using the differential Gene Test function of Monocle with q-value < 0.01, and showed by branched-heatmap images and/or scatter plot images. The CytoTRACE (Cellular (Cyto) Trajectory Reconstruction Analysis using gene Counts and Expression) analysis was performed to determine the cell subtypes located at the beginning of cell trajectory, which is a computational method that predicts the differentiation states of cells from single-cell RNA-sequencing data [34].

### 2.13. Cell Communication and Signaling Pathways

Cell communication analysis was performed using the R package CellChat [35] with default parameters. Mouse kidneys datasets were analyzed individually. The CellChatDB mouse was used for analysis. All six groups of samples were normalized together, and then each group was extracted and analyzed and compared in parallel. This is with the assumption that they were sharing cell types.

### 2.14. Statistics and Reproducibility

The details of the statistical analyses performed in this study are provided in the respective sections of the “Results” and “Methods”. For differential gene expression, a Wilcoxon Rank Sum test was used (via Seurat version 3.6.2) to assess the statistical significance of gene expression differences across cell clusters.

## 3. Results

### 3.1. Single-Cell RNA-Seq Profiling of Pkd1 Heterozygous and Pkd1 Homozygous Knockout Kidneys at Different Ages and Disease Stages

**Figure 1:**
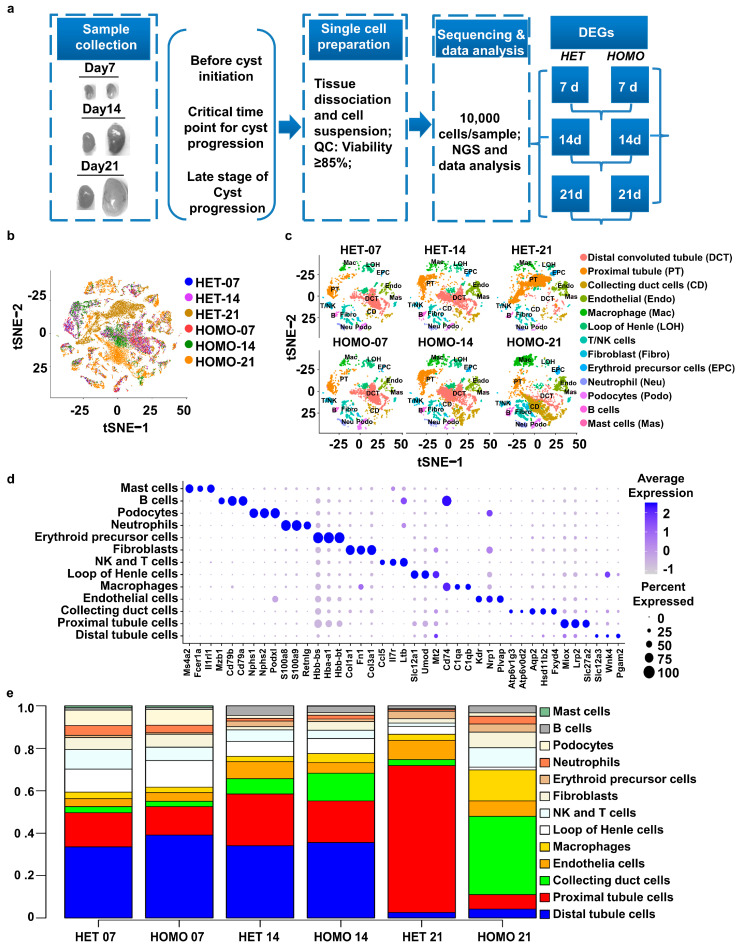
**Single-cell RNA sequencing reveals cell diversity in polycystic kidney disease.** (**a**) Workflow showing the collection of mouse kidneys, including paired HET and HOMO kidneys of a *Pkd1* mouse model. (**b**) tSNE plot showing cell distributions from six different kidney groups, including 3 *Pkd1* heterozygous deletion mice (*Pkd1* HET-07/14/21) and 3 *Pkd1* homozygous deletion mice (*Pkd1* HOMO-07/14/21). Each color represents a separate group. (**c**) tSNE plot showing the distribution of cells from six groups of kidney samples. Each color represents a cell type. (**d**) Dot plot showing expression of selected marker genes in each cell type. The size of the dot indicates the percentage of cells within a cell type in which that marker was detected, and its color indicates the average expression level. The same tSNE is plotted in (**e**), showing only cells from each sample. (**e**) Stacked barplot graph showing the percentage composition of each of the cell types in the six kidney groups. Each column adds up to 100%.

To analyze single-cell transcriptomics in *Pkd1* conditional knockout kidneys, we collected 18 kidneys from *Pkd1^flox/+^:Pkhd1*-Cre (named *Pkd1* HET hereafter, n = 3) mice and *Pkd1^flox/flox^:Pkhd1*-Cre (named *Pkd1* HOMO hereafter, n = 3) mice at postnatal days 7, 14 and 21, at which three mouse kidneys were collected in each group, as outlined in Figure 1a.

Both male and female mice were collected in each kidney group. In these *Pkd1* conditional knockout mice, *Pkd1* deletion was driven by *Pkhd1* promoter-mediated *Cre* recombinase, which is mainly expressed in collecting ducts [24,25]. The *Pkd1* HET mice developed normally with no cyst formation in the kidneys for up to just over one year old, whereas renal cysts initiated at or around postnatal days 8–9 and cysts were extensively developed at postnatal day 21 in the kidneys from *Pkd1* HOMO mice [19,36]. It has been reported that postnatal day 14 is a critical time point for determining cyst development in *Pkd1* conditional knockout mouse kidneys [37]. Thus, we collected *Pkd1* HET (control) and *Pkd1* HOMO kidneys at postnatal day 7 before cyst initiation (pre-cyst formation stage), at postnatal day 14 (critical time point/stage) and at postnatal day 21 when cysts are aggressively developing in *Pkd1^flox/flox^:Pkhd1*-Cre kidneys (late stage of cyst formation) [38].

We performed several important quality-control analyses to remove ambient RNA and doublets before further analysis (Figure S1). We clustered all cell types across the six kidney groups and produced clusters with the t-distributed stochastic neighbor embedding (tSNE) method. As shown in Figure 1b, the cells are color-coded according to their original kidney group, with each color representing cells from one of the six kidney groups.

### 3.2. Classification of Renal Cells Based on Cell Type-Specific Marker Genes across Six Groups of Pkd1 Heterozygous and Pkd1 Homozygous Kidneys at Different Ages and Disease Stages

The clusters for each kidney group were annotated by interrogating the expression of known marker genes (Figure 1c,d), as identified by differential gene expression (DGE) analysis. The expression levels of different marker genes based on an existent dataset [39] were used for the manual annotation of each cluster (Figure 1d), including Aqp2 (aquaporin 2) for collecting duct cells, Lrp2 (lipoprotein-related protein 2) for proximal tubule cells, Kdr (vascular endothelial growth factor receptor 2) for endothelial cells, Nphs1 (nephrin) and Nphs2 (podocin) for podocytes, Slc12a1 (Na-K-2Cl cotransporter) for the ascending loop of Henle [26,40], and Il1rl1 and Ms4a2 for mast cells [41]. The cell type markers and proportions of clusters of *Pkd1* HET kidneys are highly consistent with single-cell sequencing data from adult wildtype mouse kidneys [26], validating *Pkd1* HET kidneys as a control for the annotation of the clusters in *Pkd1* HOMO kidneys. We also found that proportions of clusters were highly consistent within the groups of mouse kidneys, in which the cells from three replicates were distributed evenly in all clusters (Figure S2).

We identified 13 major cell types and the percentages of each cell type across the six groups of *Pkd1* HET and HOMO kidneys (Figure 1c–e). The changes in the populations of proximal tubule cells, CD-principal cells, and CD-intercalated cells, marked with different marker genes, are also shown in feature plots (Figure S3a–c). Renal cysts originate mainly from the collecting ducts in *Pkd1* HOMO kidneys [24,25]. We found that the percentages of CD cells increased in *Pkd1* HET kidneys at day 14 compared to those in *Pkd1* HET kidneys at day 7, and then decreased in day 21 *Pkd1* HET kidneys to a percentage similar to that in day 7 *Pkd1* HET kidneys. In contrast, the percentages of CD cells steadily increased in *Pkd1* HOMO kidneys from day 7 to day 21 (Figure 1c,e), consistent with the proliferation of cystic collecting duct cells increasing from day 7 to day 21. The proportions of other cell types (clusters), including macrophages, fibroblasts, and NK cells and T cells (Figure S3d–f), as well as Loop of Henle cells, neutrophils, and podocytes (Figure S4a–c), gradually decreased in *Pkd1* HET kidneys during normal development from day 7 to day 21, but increased in *Pkd1 HOMO* kidneys from day 7 to day 21.

### 3.3. Classification of Collecting Duct Cells Based on Cell Subtype-Specific Marker Genes in Pkd1 Heterozygous and Pkd1 Homozygous Kidneys at Different Ages and Disease Stages

**Figure 2:**
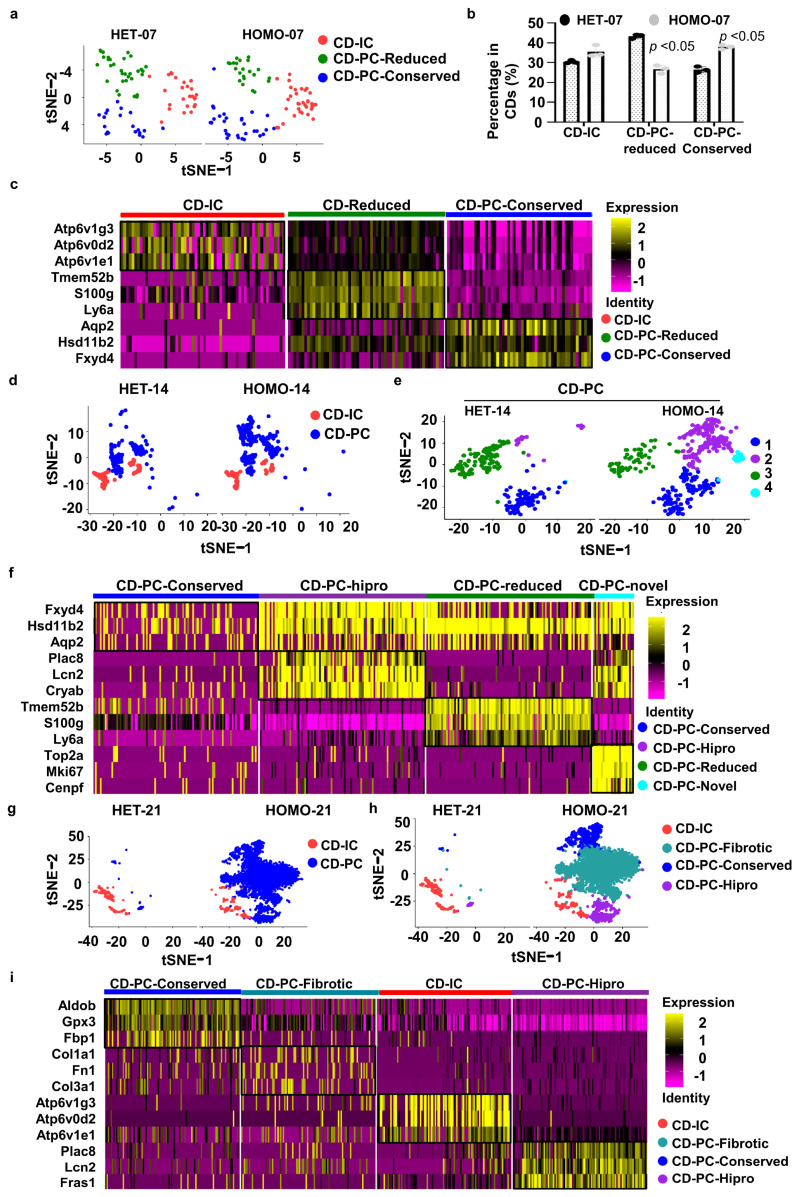
**Identification of sub-populations of collecting duct cells at different cyst formation stages of *Pkd1* heterozygous and homozygous kidneys.** (**a**–**c**) Identification and characterization of collecting duct cell subtypes from day 7 *Pkd1* HET and HOMO kidneys. (**a**) tSNE plots showing sub-clustering of collecting duct cells from day 7. (**b**) Sub-clustering compositions of collecting duct cells from day 7 comparing *Pkd1* HET and HOMO kidneys. (**c**) Heatmap shows specific markers for each subtype. Each column represents a cell, and each row represents a gene. (**d**–**f**) Identification and characterization of collecting duct cell subtypes from day 14 *Pkd1* HET and HOMO kidneys. (**d**) tSNE plots showing sub-clustering of collecting duct cells (CD-IC and CD-PC). (**e**) Sub-clustering compositions of collecting duct principal cells from day 14 comparing *Pkd1* HET and HOMO kidneys. (**f**) Heatmap shows specific markers for each subtype of collecting duct principal cells from day 14 *Pkd1* HET and HOMO kidneys. (**g**–**i**) Identification and characterization of collecting duct cell subtypes from day 21 *Pkd1* HET and HOMO kidneys.

First, because the *Pkd1* gene was deleted primarily in collecting duct cells in *Pkd1* HET and *Pkd1* HOMO kidneys, we further sub-clustered collecting duct cells from those kidneys using tSNE analysis. We identified three sub-clusters of CD cells in day 7 *Pkd1* HET and HOMO kidneys (Figure 2a), including CD intercalated cells (named CD-IC cells hereafter) (marked with Atp6v1g3 and Atp6v0d2) and CD principal cells (named CD-PC cells hereafter). CD-PC cells were further sub-clustered into CD-PC-Conserved cells (marked with Aqp2 and Hsd11b2), which were relatively conserved between day 7 *Pkd1* HET and *Pkd1* HOMO kidneys, and CD-PC-Reduced cells, which were decreased in day 7 *Pkd1* HOMO kidneys compared to *Pkd1* HET kidneys, resulting in its designation “reduced” (Figure 2a,b). With the reduction in the CD-PC-Reduced cell population, there was a concomitant increase in the CD-IC and CD-PC-Conserved cell populations in day 7 *Pkd1* HOMO kidneys (Figure 2b), but there was almost no difference in these CD cell populations in day 7 *Pkd1* HET kidneys versus *Pkd1* HOMO kidneys, suggesting that the CD-IC and CD-PC-Conserved cells were increased before postnatal day 7 even though no cysts were detected in day 7 *Pkd1* HOMO kidneys.

Second, with the same parameters used for clustering day 7 kidneys, we could directly differentiate a CD-IC cluster from a CD-PC cluster without sub-clustering CD cells in day 14 kidney samples (Figure 2d). By sub-clustering the CD-PC cells, we identified two extra CD-PC sub-clusters in *Pkd1* HET-14 and *Pkd1* HOMO-14 kidneys in addition to the CD-PC-Conserved and CD-PC-Reduced sub-clusters identified in day 7 kidneys (Figure 2e): the CD-PC-Hipro (for high proliferation) sub-cluster, which was marked by Plac8, Lcn2 and Cryab (Figure 2f), and the CD-PC-Novel sub-cluster, which was hardly seen in CD-PC cells in *Pkd1* HET-14 kidneys, but was identified in *Pkd1* HOMO-14 kidneys (Figure 2f).

Third, with the same parameters used for clustering day 7 and day 14 kidneys, we could also directly differentiate a CD-IC cluster from a CD-PC cluster in day 21 kidney samples without sub-clustering CD cells (Figure 2g). We identified three sub-clusters of CD-PC cells, including CD-PC-Conserved, CD-PC-Hipro, and CD-PC-Fibrotic cells in *Pkd1* HET-21 and HOMO-21 kidneys (Figure 2h), and these were markedly increased in *Pkd1* HOMO-21 kidneys compared to *Pkd1* HET-21 kidneys. We found that the CD-PC-Conserved cells in day 21 *Pkd1* HOMO kidneys not only expressed marker genes characteristic of this population, but also expressed high levels of metabolism-related genes, including Aldob, Gpx1, Gpx3, Glyat, Gsta2, and Hbb (Figure 2i), which are related to oxygen metabolism and mitochondrial function, suggesting a high metabolic state for this cell subtype at this late stage of cyst progression. CD-PC-Fibrotic cells were the dominant sub-cluster in *Pkd1* HOMO-21 kidneys compared to *Pkd1* HET-21 kidneys. These CD-PC-Fibrotic cells not only expressed CD-PC markers, but also expressed high levels of fibrotic genes, including Col1α1, Col1α2 and Col3α1, among other fibrotic genes (Figure 2i).

**Figure 3:**
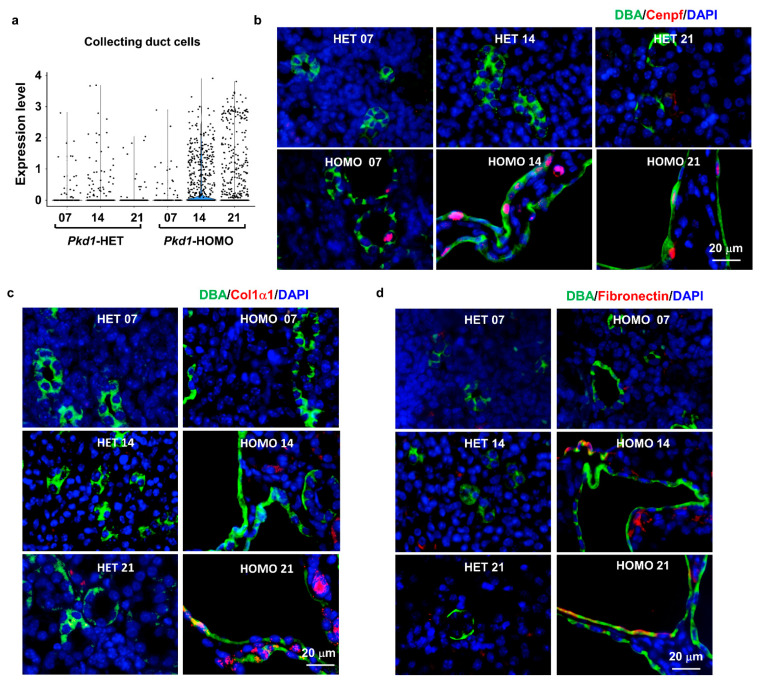
**Expression pattern of transitional cell marker genes.** (**a**) Violin plots showing the expression of Cenpf in collecting duct cells. (**b**) Representative immunofluorescence staining for Cenpf in *Pkd1* heterozygous and homozygous knockout mouse kidneys. Green color labels DBA and red color indicates Cenpf. (**c**,**d**) Representative immunofluorescence staining for Col1α1 (**c**) and fibronectin (**d**) in *Pkd1* heterozygous and homozygous mouse kidneys. Green color labels DBA and red color indicates Col1a1 and fibronectin, respectively.

It has been reported that that postnatal day 14 is a critical stage for both kidney development and cyst progression [37]. In day 14 kidneys, we identified a novel collecting duct cell subtype, named CD-PC-novel, with a high expression of genes related to cell proliferation, DNA breaks for DNA replication and both cell proliferation and metastasis, including antigen KI-67 (Mki67 or Ki67), DNA topoisomerase II alpha (Top2a) and centromere protein F (Cenpf) [42,43,44]. Cenpf is involved in chromosome segregation during cell division, which also plays a role in the orientation of microtubules to form cellular cilia [45]. The expression of Cenpf was found upregulated in collecting duct cells from *Pkd1* HOMO kidneys compared to that in age-matched *Pkd1* HET kidneys at a single-cell resolution (Figure 3a), and this was confirmed by immunofluorescence staining with Cenpf antibody and co-staining with collecting duct cell marker, DBA (Figure 3b). We noticed that the expression of Cenpf was higher in day 14 compared to day 21 *Pkd1* HOMO kidneys, which is consistent with the single cell analysis. Because the CD-PC-novel cell population was only identified in day 14 kidneys, especially in *Pkd1* homozygous kidneys, the upregulation of Cenpf may be one of the critical factors in promoting cyst progression at this time point. In addition, to validate the existence of CD-PC-Fibrotic cells in *Pkd1* mutant mouse kidneys, we performed immunofluorescence staining with fibrotic markers, including Col1a1 and Fibronectin. We found that the expression levels of both markers were gradually increased in cyst-lining epithelial cells in *Pkd1* knockout kidneys, especially at postnatal day 21, while they were only localized in the interstitium in *Pkd1* heterozygous mouse kidneys and early-stage *Pkd1* mutant mouse kidneys (Figure 3c,d).

Last, we also analyzed collecting duct cells during *Pkd1* HET kidney development and *Pkd1* HOMO cyst progression from postnatal day 7 to day 21. We identified three sub-clusters of CD cells in *Pkd1* HET-7, -14 and -21 kidneys, including CD-IC, CD-PC-Conserved, and CD-PC-Reduced cells (Figure S5a), which were identified by different marker genes, as shown in Figure S5b. However, via this analysis, we could not identify sub-clusters of CD-PC-hipro or CD-PC-Novel cells in the *Pkd1* HET kidneys.

**Figure 4:**
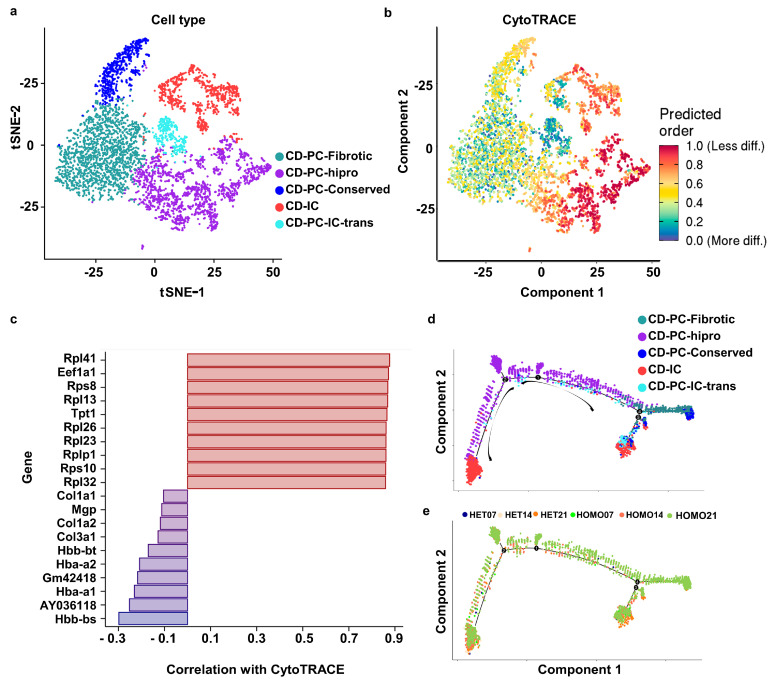
**CytoTRACE and Monocle cell trajectory analysis of collecting duct cell differentiation from *Pkd1* heterozygous and homozygous knockout kidneys day 7 (pre-initiation stage), day 14 (progressive stage) and day 21 (late stage).** (**a**) tSNE visualization of renal collecting duct cells merged from the six kidney groups, which are colored by transcriptional cluster, with each color representing a different cell subtype. (**b**) Projection of the results from CytoTRACE on the integrated data of collecting duct cells by Seurat. A higher score indicates less differentiated states. (**c**) The top 10 (less differentiated; red) and bottom 10 (most differentiated; blue) genes in this dataset based on their correlation with CytoTRACE. (**d**,**e**) Cell trajectory maps of collecting duct cells highlighting the contribution of cells coming from each subtype (**d**) and each group (**e**). Arrows show the trajectory initiated from CD-PC-hipro cells (**d**). Each color in d represents one cell subset of collecting duct cells. Each color in (**e**) represents the original kidney group of collecting duct cells.

When we compared the CD cells among all three stages of *Pkd1* HOMO kidneys, we also identified four sub-clusters of CD cells in those kidneys, including CD-IC, CD-PC-Conserved, CD-PC-hipro and CD-PC-Fibrotic cells (Figure S5c), as identified by different marker genes (Figure S5d). We formed a subset of the CD-PC collecting duct cells from day 14 *Pkd1* HET and HOMO kidneys, and we identified a CD-PC-novel cell subset, as it is the novel population dominant in day 14 *Pkd1* HOMO, but not in day 14 *Pkd1* HET, kidneys. However, when we subset the collecting duct cells, including CD-IC, from all six groups, three *Pkd1* HET groups or three *Pkd1* HOMO groups, certain cell subtypes might not be present when using the same parameters (Figure 2, Figure S5, Figure 4). This is because we knew that the cell subsets were identified according to the cell heterogeneity and similarity between cells, and calculated by the parameter termed resolution in the single-cell analysis program.

### 3.4. Reconstruction of Collecting Duct Cell Trajectories during Cyst Development in Pkd1 Homozygous Knockout Kidneys

During tissue development and throughout life, cells can be transited from one functional “state” to another. Cells in different states undergo a process of transcriptional re-configuration, with some genes being silenced and others being activated, thus driving cell transition between states. These transient states are often hard to characterize in purified cells since these cells are in more stable endpoint states. To prove the cell transition and identify genes that driving cell transition between states, we performed CytoTRACE (Cellular (Cyto) Trajectory Reconstruction Analysis using gene Counts and Expression) and Monocle analysis.

To analyze the cell transition between connecting duct cell subtypes during the development and disease progression, we subset the connecting duct cells across all the stages (days 7, 14 and 21 of *Pkd1* HET and HOMO kidneys). We identified five subtypes of collecting duct cells, CD-PC-Fibrotic, CD-PC-hipro, CD-PC-Conserved, CD-IC and CD-PC-IC-trans (Figure 4a). To determine the cell subtypes located at the beginning of cell trajectory, we performed the CytoTRACE analysis. We found that the CD-PC-hipro cells were less differentiated, while CD-PC-Fibrotic and CD-PC-IC-trans cell subtypes were more differentiated, suggesting that CD-PC-hipro cells were at the beginning state of collecting duct cells, while some CD-PC-Fibrotic and most of the CD-PC-IC-trans cells were at the end states of cell transition of collecting duct cells (Figure 4b). Furthermore, the genes associated with differentiation can be predicted based on their correlation with CytoTRACE. We identified the top 10 (less differentiated; *red*) and bottom 10 (most differentiated; *blue*) genes in this dataset based on their correlation with CytoTRACE (Figure 4c). The genes associated with less differentiated cells were mainly from the ribosomal protein (RP) gene family, including both small (RPS) and large (RPL) subunits, supporting the high potential of proliferation features of these cells, while the genes associated with more differentiated cells were fibrotic markers, such as Col1α1, Col1α2 and Col3α1. The expression of Col1α1 was confirmed by the immunostaining in kidneys (Figure 3c). We also ordered collecting duct cells with Monocle program according to their cell sub-types and their original groups (Figure 4d,e). We found that CD-PC-hipro cells (Figure 4a,d, in blue violet color) might initiate the transition, which is consistent with the prediction by CytoTRACE analysis, whereas we noticed that these cells were not ordered in a time series manner (Figure 4d). Collecting duct cells in day 21 *Pkd1* HOMO kidneys were the dominant cell population with high heterogeneity compared to collecting duct cells from other groups. Thus, they were widely distributed to all the states of cell trajectory analysis (Figure 4e).

### 3.5. Systematic Analysis of Cell Type-Specific Differentially Expressed Genes (DEGs) Associated with PKD-Related Traits

**Figure 5:**
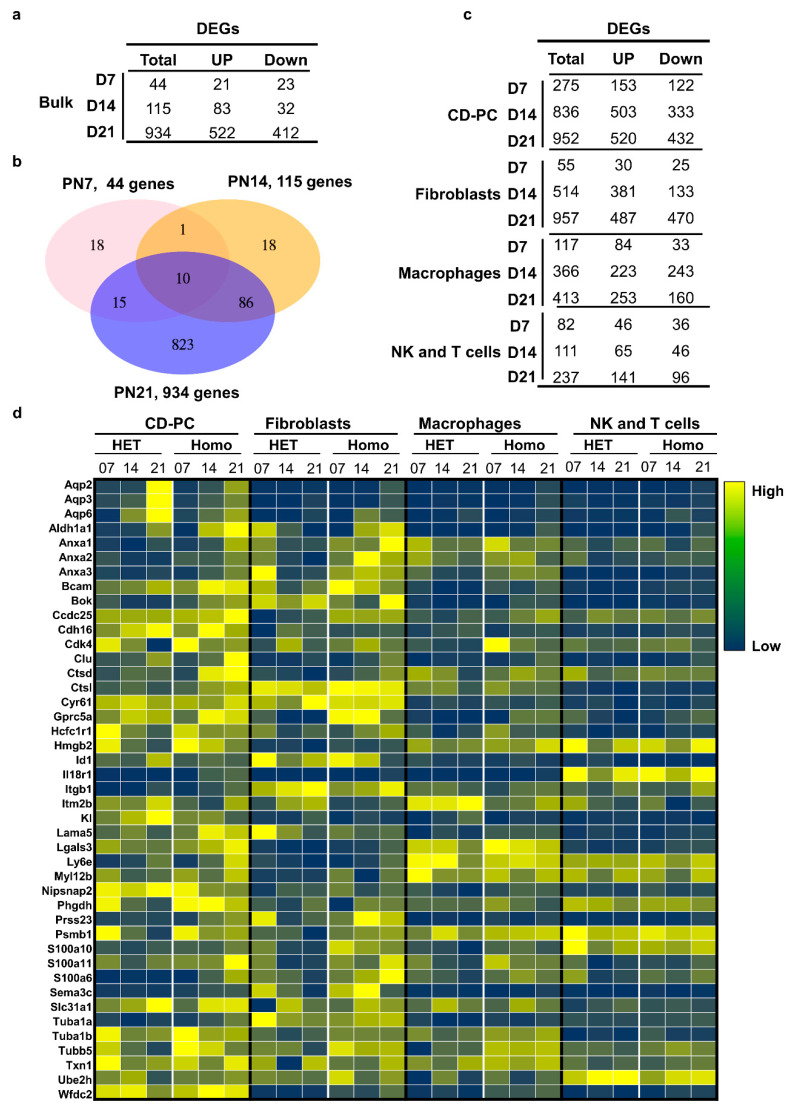
**Single-cell resolution identified higher gene expression viability within *Pkd1* heterozygous and homozygous knockout kidneys than bulk analysis.** (**a**) Graph showing DEG counts identified from bulk RNA analysis for each stage (log2 (mean gene expression in *Pkd1* HOMO/mean gene expression in *Pkd1* HET) > 0.25, Poisson mixed-model FDR < 0.05). (**b**) Venn diagram showing the overlaps of differentially expressed genes at the three stages. (**c**) DEG counts identified by single-cell RNA-seq analysis for each cell type (log2 (mean gene expression in *Pkd1* HOMO/mean gene expression in *Pkd1* HET) > 0.25, Poisson mixed-model FDR < 0.05). (**d**) Heatmap showing the top DEGs from collecting duct cells, fibroblasts, macrophages, and T/NK immune cells in day 7, 14 and 21 *Pkd1* heterozygous and homozygous kidneys.

Given the complexity and heterogeneity of *Pkd1*-mutant kidneys at these different stages, we next aimed to quantify the association between gene expression profiles in specific cell types and the variability in the pathological traits at each of the three stages of *Pkd1* HOMO kidneys (day 7, pre-cyst; day 14, critical time point for cyst formation; day 21, aggressive cyst formation). We focused on four major cell types, including collecting duct cells, macrophages, fibroblasts, and NK/T cells. We found that, when the cell type was designated, even at day 7, the numbers of DEGs identified in a specific cell type in *Pkd1* HOMO kidneys versus *Pkd1* HET kidneys were significantly increased, as compared with the bulk RNA-seq analysis (Figure 5a–c). The DEGs in these four cell types at days 7, 14 and 21 *Pkd1* HOMO kidneys versus *Pkd1* HET kidneys are listed in Supplemental Tables S1–S12. We have highlighted the top DEGs in CD-PC, fibroblasts, macrophages, and NK/T cells at different stages of *Pkd1* HOMO kidneys versus those in *Pkd1* HET kidneys (Figure 5d). We have also identified the top DEGs in collecting duct cells (CD-PC and CD-IC) at different stages (Figure S6). Our results suggest that bulk RNA analysis only provides global clues of upregulated or downregulated genes in whole kidneys, whereas single-cell RNA profile analysis could tell us about the changes in specific genes in the main cell types.

### 3.6. Cell-to-Cell Communication Network Inference Reveals Signaling “Senders” and “Receivers” during Kidney Development and Cyst Initiation and Progression

**Figure 6:**
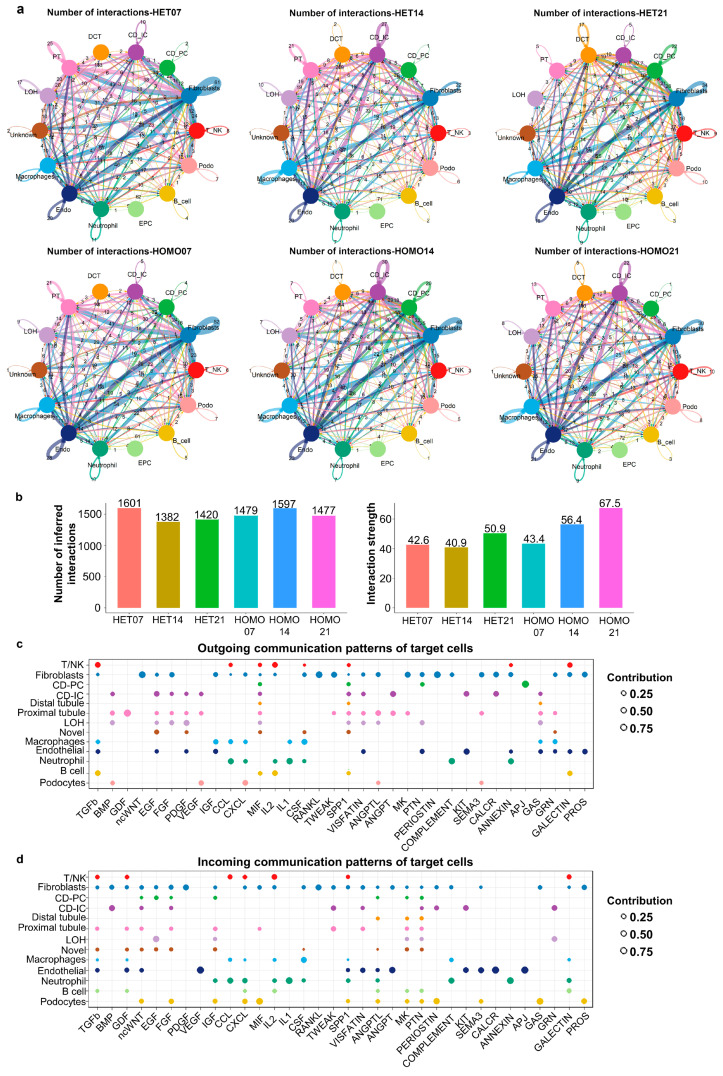
**Global analysis of cell-to-cell communications in *Pkd1* heterozygous and homozygous kidneys.** (**a**) Circle plots showing the numbers and strengths of interactions in each kidney group individually, in which each number represents the ligand–receptor pairs between two cell types in the same kidney. The round loops along with cell type represent the interactions within the same cell type. (**b**) Graphs showing numbers (left) and strengths (right) of interactions in each group. (**c**,**d**) The dot plot showing the outgoing signaling patterns of secreting cells (**c**) and incoming signaling patterns of targeting cells (**d**) in *Pkd1* HET-7 kidneys. The dot size is proportional to the contribution score computed from pattern recognition analysis. A higher contribution score implies the signaling pathway is more enriched in the corresponding cell group.

In general, renal cells should not function independently, and instead they should communicate with each other by transmitting and receiving signals within their environment. To establish a cell-to-cell communication network in normal and PKD kidneys, we used a newly developed mathematical modeling method, called CellChat, which quantitatively infers intercellular communication networks using mass action models and enables the visualization of cellular interactions [35]. With CellChat analysis, first, we established a global cell-to-cell communication networks among most of the renal cell types, including collecting duct cells, macrophages, fibroblasts, T/NK cells, etc., by counting the number of interactions (represented by a “line” connection between two cell types) and the interaction strength (represented by the “line” weights) using circle plots according to the ligand–receptor pairs in day 7, 14 and 21 *Pkd1* HET and HOMO kidneys, respectively (Figure 6a). The numbers and strengths of those interactions in each kidney group were summarized (Figure 6b); the numbers of cell-to-cell interactions are higher in *Pkd1* HET-7 and *Pkd1* HOMO-14 kidneys (1601 and 1597, respectively) than in the other four kidney groups, whereas the strengths of those interactions were gradually increased in *Pkd1* HOMO kidneys from day 7 to day 21, which might have been mediated by an increase in the expressions of either ligands, receptors or both in *Pkd1* HOMO kidneys during cyst progression.

CellChat could quantitatively measure ligand–receptor networks to predict key incoming and outgoing signals for specific cell types by leveraging pattern recognition approaches. For example, in *Pkd1* HET-07 kidneys, each type of cell could be the secreting cell (signaling senders), which could release different cytokines or ligands (Figure 6c), and each cell type could also be the targeting cells (signaling receivers) when the receptors on these cells are targeted by the ligands released from the same types of cells or other cells (Figure 6d). The ligand–receptor-mediated communications among different cell types should contribute to the development of *Pkd1* HET-7 kidneys.

Postnatal day 14 is a critical time point for cyst progression in *Pkd1* conditional knockout mice. In *Pkd1* HOMO-14 kidneys, each cell type could also be the secreting cells (signaling senders) (Figure S7a, *left*) and the targeting cells (signaling receivers) (Figure S7a, *right*) of the factors listed on the left side of Figure S7a. For example, if CD-PC cells are the sources (senders), the factors (ligands) secreted form those cells might bind to different receptors on targeting cells (Figure S7b).

**Figure 7:**
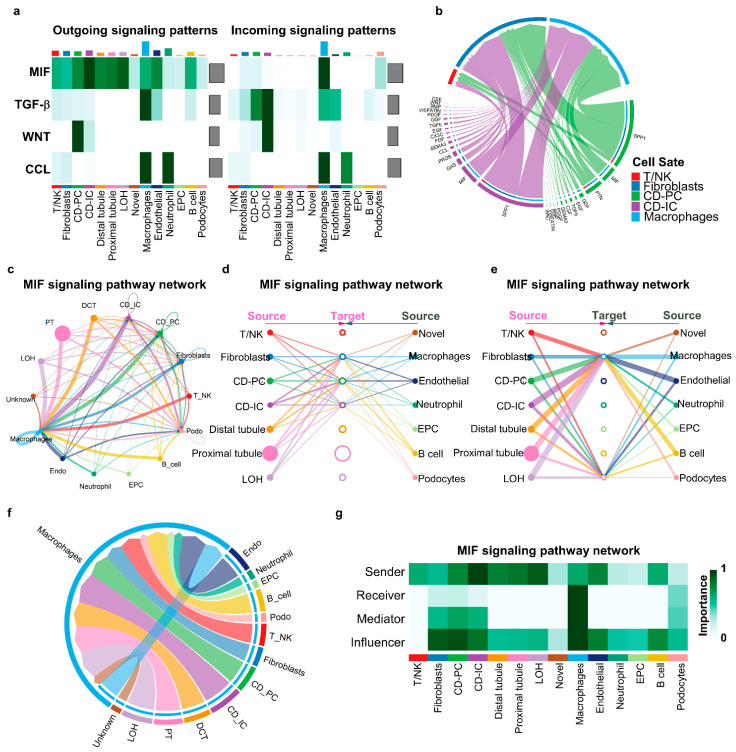
**Cell-to-cell communications in day 14 *Pkd1* homozygous kidneys as analyzed by CellChat program**. (**a**) Heatmaps of MIF, TGF-β, WNT and CCL signals contributing mostly to outgoing or incoming signaling of certain cell groups. (**b**) Chord diagram showing all the significant interactions (L–R pairs) between different cell types. (**c**) Circle plots showing the interactions and strengths among different cell types. (**d**,**e**) Hierarchical plot show the inferred intercellular communication network for MIF signaling. Solid and open circles represent source and target cell types, respectively. Circle sizes are proportional to the number of cells in each cell type. Edge colors of middle circles are consistent with the signaling source. (**f**) Chord diagram showing the network of MIF signaling pathways mediated only by MIF-CD74/CD44 in different cell types. (**g**) Heatmap shows the relative importance of each cell type as sender, receiver, mediator and influence, based on the computed four network centrality measures of MIF signaling.

We found that macrophage migration inhibitory factor (MIF) could be highly secreted by almost all types of cells in *Pkd1* HOMO-14 kidneys (Figure 7a, left), whereas its main targeting cells (receivers) are macrophages, CD-PC cells, fibroblasts and podocytes (Figure 7a, right). With the comparison of the factors (ligands) significantly secreted from

CD-IC and CD-PC cells and received by T/NK, fibroblasts and macrophages, we found that CD-IC and CD-PC cell-secreted MIF could strongly target macrophages and fibroblasts, as shown in the Chord diagram (Figure 7b). However, if all cell types were included in this analysis, MIF could mediate the communications among all cell types in *Pkd1* HOMO-14 kidneys, as shown in the circle plot (Figure 7c). If we set all of the 14 identified cell types in *Pkd1* HOMO-14 kidneys as resource cells of MIF and set the 7 cell types listed on the left side of Figure 7d as potential targeting cells, the hierarchical plots indicate that the MIF released for all 14 cell types could target CD-IC and CD-PC cells and fibroblasts (Figure 7d), whereas if the other 7 cell types listed on the right of Figure 7e were set as potential targeting cells, the hierarchical plots indicate that the MIF released for all 14 cell types could target macrophages and podocytes, in which macrophages are strongly targeted compared to podocytes (Figure 7e). These results suggest that all cell types in *Pkd1* HOMO-14 kidneys are possible sources of MIF, whereas only macrophages, fibroblasts, CD-IC and CD-PC cells, and podocytes are MIF-targeting cells with different targeting strengths (the line width between cells) (Figure 7d,e). We further found that the interactions of all cell types with macrophages were mediated by the binding of MIF with its receptors, CD74/CD44 (Figure 7f). In addition to the sender and receiver for MIF signaling, we also identified the cell types as mediators and influencers for MIF signaling-mediated intercellular communications, according to the relative importance of each cell type based on an algorithm, which was named “centrality measure” (Figure 7g). Furthermore, we also identified the interactions between different cell subtypes of collecting duct cells mediated by MIF signaling. MIF and its receptors CD74/CD44 were expressed in cell subsets of collecting duct cells (Figure S8a). We found that all five CD subsets could be resource cells, while CD-PC-hipro cells were the only receiver cells for MIF-CD44/CD74 signaling (Figure S8b). The conclusion from this analysis is supported by our recent study, which found that MIF played an important role in regulating different signaling pathways, and the inhibition of MIF delays cyst growth in *Pkd1* mutant mice [19].

### 3.7. Potential Mechanisms for Collecting Duct Cells to Affect Neighboring Cells during Cyst Initiation and Progression

Cysts are initiated from collecting duct cells in *Pkd1^flox/flox^: Pkhd1*-Cre kidneys. A key question needing to be addressed is how *Pkd1* mutations in collecting duct cells might affect gene expression in neighboring cells and even distant cells. Direct cell-to-cell contacts between *Pkd1* mutant collecting duct cells and neighboring cells may influence the biology and function of adjacent cells. However, *Pkd1* mutant collecting duct cells may also affect adjacent and more distant cells through secreted factors. Among the possible factors secreted by *Pkd1* mutant collecting duct cells are cytokines. We propose that the expression of cytokines and receptors on *Pkd1* mutant collecting duct cells makes these cells “signal sending” cells (if their cytokines are secreted) and “signal receiving” cells (if these receptors bind with cytokines secreted by neighboring cells).

**Figure 8:**
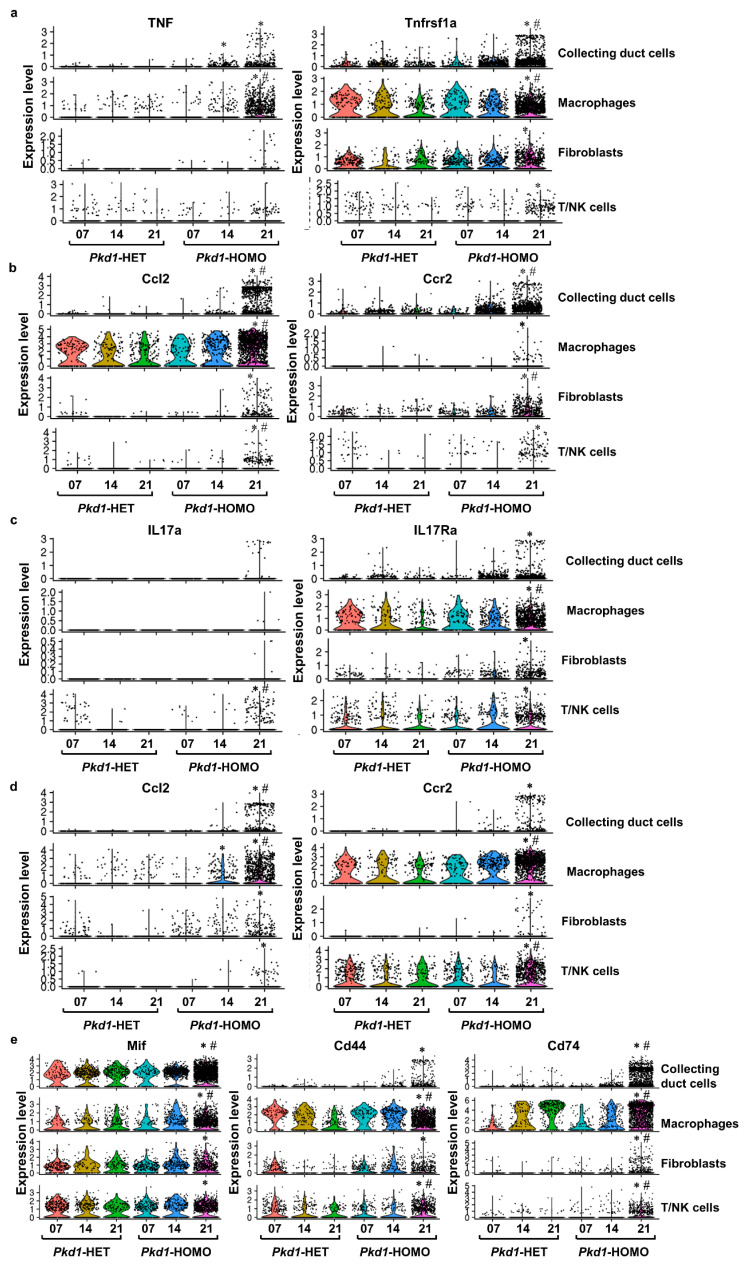
**The expression of cytokines and their corresponding receptors was changed in collecting duct cells, macrophages, immune cells and fibroblasts.** (**a**) Violin plots showing TNF and its receptor TNF RI’s dynamic expression in four types of cells during PKD progression. (**b**) Violin plots showing IL1b and its receptor IL1r1’s dynamic expression in four types of cells during PKD progression. (**c**) Violin plots showing IL17a and its receptor IL17Ra’s dynamic expression in four types of cells during PKD progression. (**d**) Violin plots showing CCL2 and its receptor Ccr2’s dynamic expression in four types of cells during PKD progression. (**e**) Violin plots showing MIF and its receptors CD74 and CD44’s dynamic expression in four types of cells during PKD progression. * represents the comparison between *Pkd1* HOMO kidneys and age-matched *Pkd1* HET kidneys (*p* < 0.05). ^#^ represents the comparison between *Pkd1* HOMO 21 kidneys and *Pkd1* HOMO 7 and 14 kidneys, respectively (*p* < 0.05), as calculated by a one-way ANOVA test.

To support this hypothesis, we found that the expression of some cytokines was increased in collecting duct cells, macrophages, fibroblasts and immune cells, and their corresponding receptors were also upregulated in either the same cell type or other cell types during cyst progression (Figure 5d). In particular, we identified correlations between TNF and its receptor Tnfsf1a; Il1b and its receptor Il1r1; IL17a and its receptor IL17ra; Ccl2 and its receptor Ccr2; and MIF and its receptors CD74 and CD44 among these four cell types (Figure 8). For example, TNF was upregulated as early as day 7, and was upregulated in macrophages at day 14 and 21. It was not significantly upregulated until day 21 in collecting duct cells and fibroblasts, while the upregulation of its receptors was found in fibroblasts first, and did not increase in collecting duct cells until day 21 (Figure 8a). IL1b was expressed at high levels in macrophages, but gradually increased in the other three cell types, whereas the expression of its receptor IL1r1 gradually increased in these four cell types (Figure 8b). The expression of IL17a and its receptor IL17ra, as well as Ccl2 and its receptor Ccr2, was upregulated in all four of these cell types at day 21 (Figure 8c,d). Importantly, the expression of MIF was high at the three stages of collecting duct development, whereas its receptors, CD74 and CD44, did not become highly upregulated in collecting duct cells until day 21 (Figure 8e). We also noted a differential expression pattern for CD74 in NK/T cells at days 7, 14 and 21, and in fibroblasts at day 21 (Figure 8e). We previously reported that MIF promotes cyst growth in ADPKD [19]. We found that the expression of MIF and its receptors CD74/CD44 was increased at both mRNA and protein levels in *Pkd1* HOMO mouse kidneys compared to *Pkd1* HET mouse kidneys (Figure S8c–f). Taken together, we propose that CD cells secrete MIF and macrophages secrete TNF, which bind to their receptors on collecting duct cells and other cell types to regulate the expressions of other cytokines and cytokine-mediated signaling pathways, forming a positive feedback loop to promote cell proliferation and cyst progression. Other cytokines and their receptors might function in a similar way, which might be a key mechanism in understanding how *Pkd1* deletion in CD cells affects other adjacent or distant cells, including other renal epithelial cells, immune cells and fibroblasts.

**Figure 9:**
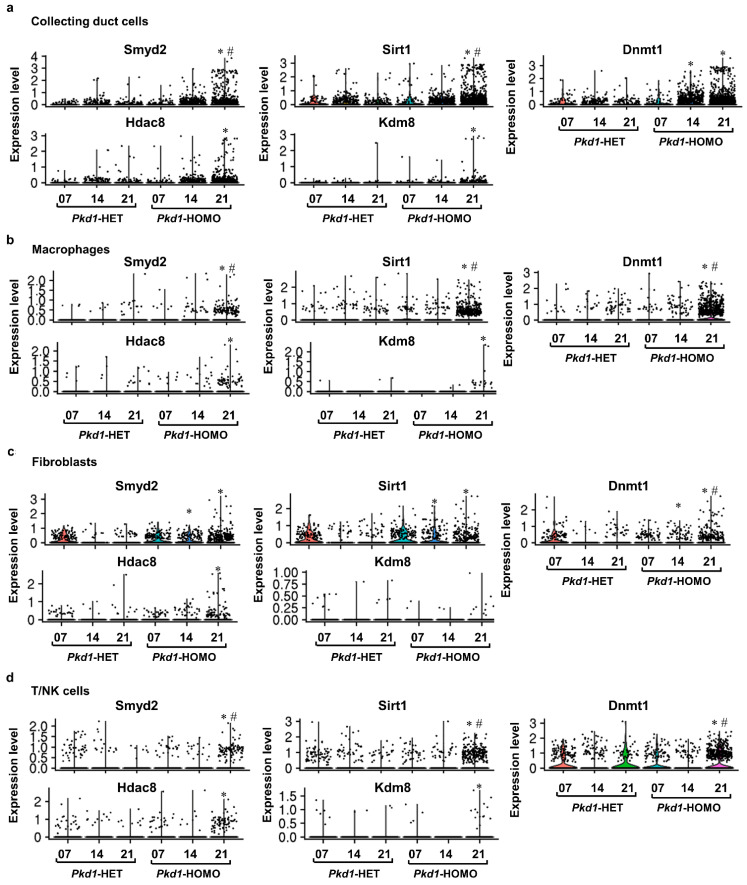
**The expression of epigenetic factors was changed in collecting duct cells, macrophages, immune cells and fibroblasts.** (**a**–**d**) Violin plot showing the upregulations of Smyd2, Sirt1, Dnmt1, Hdac8 and Kdm8 in collecting duct cells (**a**), macrophages (**b**), fibroblasts (**c**) and T/NK immune cells (**d**) across days 7, 14 and 21 in *Pkd1* HET and HOMO mouse kidneys. * represents the comparison between *Pkd1* HOMO kidneys and age-matched *Pkd1* HET kidneys (*p* < 0.05). ^#^ represents the comparison between *Pkd1* HOMO 21 kidneys and *Pkd1* HOMO 7 and 14 kidneys, respectively (*p* < 0.05), as calculated by a one-way ANOVA test.

Another question needing to be addressed is how the cytokines binding to their receptors affect gene expression in these adjacent or distant cells. Epigenetic regulators exert their functions as transcription factors to regulate the expressions of lots of genes in many signaling pathways. We hypothesize that one of the possible mechanisms by which secreted cytokines affect neighboring cell biology is to affect the expression of epigenetic regulators. To investigate this, we compared the DEGs of epigenetic regulators in different cell types at different stages of *Pkd1* HOMO kidneys. We found a differential expression pattern for the epigenetic regulators, including Sirt1, Smyd2, DNMT1, HDAC8 and Kdm8, in these cell types at different stages of *Pkd1* HOMO kidneys compared to age-matched *Pkd1* HET kidneys (Figure 9). The spatiotemporal regulation of epigenetic mechanisms in these four cell types may be trigged by cytokines in *Pkd1* HOMO kidneys.

## 4. Discussion

scRNA-seq can facilitate a greater understanding of the changes in gene expression in defined cell types in polycystic kidneys. In this study, we sampled *Pkd1* mutant mouse renal cells, fully characterized cell diversity, and elucidated functional changes. The analysis of kidneys where *Pkd1* expression has been eliminated mainly in collecting duct (CD) cells allows questions to be explored about the role of *Pkd1* loss in CD epithelial cells in driving disease throughout the kidney. First, we generated scRNA-seq data in kidneys from *Pkd1* conditional mice collected before cyst initiation, during cyst progression, and at late-stage cystic disease, plus aged-matched *Pkd1* heterozygous kidneys, and produced an atlas of renal cell types based on transcriptional signatures in these kidneys. We characterized cell diversity in the kidneys and identified and validated novel collecting duct cell subtypes. Secondly, we inferred cell type compositions and allele-specific gene expression in each cell type by integrating scRNA-seq and bulk RNA-seq data generated in parallel from cystic and normal kidneys to construct gene modules to recapitulate biological pathways. Third, we investigated interactions in the cystic microenvironment between *Pkd1* mutant cystic cells and non-cystic cells, which provided a detailed molecular description of the environment in which cysts grow and identified potential targets for disrupting detrimental cystic cell–microenvironment interactions. Finally, we proposed a novel theory to address how *Pkd1* mutant renal epithelial cells affect their neighboring cells based on the expression profiles of cytokines secreted by *Pkd1* mutant renal epithelial cells (donor cells) that alter the state of recipient cells, such as macrophages, T cells, and fibroblasts in the microenvironment. In particularly, cytokines secreted by *Pkd1* mutant renal epithelial cells may affect gene expression profiles in recipient cells via epigenetic mechanisms, which may participate in disease pathogenesis. By detailed characterization of the cell atlases of these *Pkd1* mutant kidneys, our studies have provided novel insights into cell-type specific functions that can power the therapeutic targeting of ADPKD. In addition, the resulting resource provides a single-cell view of transcriptional alterations associated with ADPKD pathology, and reveals cell-type-specific and shared gene expression changes, disease-associated cellular subpopulations, and cyst microenvironment-mediated transcriptional responses.

In constructing a comprehensive, spatiotemporally resolved gene expression atlas of PKD kidneys at the single-cell resolution, the *Pkd1* mouse model has several advantages compared to late-stage ADPKD human kidneys, including small size, the accessibility of different disease stages (time points), an inbred genetic background, and genetic manipulability. Because of the large size and highly fibrotic character of human ADPKD kidneys, it is impossible to generate a whole-kidney single-cell suspension. The whole-kidney profiling of PKD mouse kidneys will allow the generation of a comprehensive atlas of cell types in late-stage PKD kidneys. In addition, the PKD mouse model allow us to measure the changes in gene expression profiles in each cell type in *Pkd1* homozygous kidneys at different ages and disease stages. Thus, whole mouse kidney single-cell transcriptional atlases of *Pkd1* homozygous kidneys will represent an important step for detailed investigations of the roles of specific genes and regulatory pathways in cyst initiation and development.

Our comprehensive atlas of *Pkd1* heterozygous and *Pkd1* homozygous mouse kidneys offers a powerful resource for investigating the molecular underpinnings of cell-fate decisions during a key period (postnatal day 7 to day 21) of *Pkd1* heterozygous kidney development and *Pkd1* homozygous knockout kidney cyst progression. We exploited this resource to investigate three specific cyst development phenomena: (1) the emergence of novel collecting duct cell subtypes in cystic kidneys, (2) the trans-differentiation of collecting duct cells to those that contribute to the composition of the renal cysts, and (3) the interactions between collecting duct cells and immune cells and fibroblasts that contribute to renal cyst progression. We also used our atlas as a reference for the analysis of *Pkd1*^−/−^ collecting duct cells to identify a transcriptional state unique to *Pkd1*^−/−^ collecting duct cells in cystic kidneys, and highlighted the key differentially expressed genes that are critical for cyst progression.

One of the key observations of our scRNA-seq analysis of *Pkd1* conditional knockout mouse kidneys was the identification of different collecting duct cell subtypes and the transitions between those cell subtypes. The CD-PC-Fibrotic cells were the dominant cell subtype in *Pkd1* HOMO-21 kidneys compared to *Pkd1* HET-21 kidneys, which may guide us to further understand the epithelial–mesenchymal transition (EMT) process, a widely accepted mechanism by which injured renal tubular cells transform into mesenchymal cells that contribute to the development of fibrosis in chronic renal failure. We also found that transcriptional alterations seemed to stem from changes in cell state, with certain collecting duct cell subpopulations more readily captured in PKD pathology.

Another important result of our scRNA-seq analysis was the identification of changes in gene expression in cells adjacent to *Pkd1* mutant collecting duct cells, including in macrophages, T cells, and fibroblasts during cyst initiation and progression. Previous studies at the bulk tissue-level have implicated the abnormal upregulation and infiltration of cytokines and chemokines as one of the mechanisms that therapeutically mitigates ADPKD [46,47,48]. With scRNA-seq analysis, we identified a correlation between the expression of cytokines and their receptors in *Pkd1* mutant collecting duct cells and neighboring cells. A possible scenario is that the upregulation of some cytokines in *Pkd1* mutant collecting duct cells in cystic kidneys during cyst initiation and progression leads to increases in the secretion of those cytokines, and these secreted cytokines may then bind to their receptors on neighboring cells, including macrophages, T cells and fibroblasts, to affect the biology and function of those cells, including the expression of cytokines and cytokine receptors as well as epigenetic regulators in these recipient cells. The upregulation of cytokines in neighboring cells may also result in their secretion via dysregulated exocytosis processes, which then bind and activate receptors on *Pkd1* mutant collecting duct cells to generate a positive feedback loop between *Pkd1* mutant collecting duct cells and neighboring cells in cystic kidneys.

Renal collecting duct cells are normally responsible for the regulation of blood pressure and body fluid composition, and the collecting duct is the major site of renal cyst formation in ADPKD kidneys [49,50,51]. It is necessary, therefore, to identify the cellular subtypes in normal vs. *Pkd1*-mutant collecting ducts, and to systematically investigate the gene expression profiles (i.e., transcriptomes) of those cellular subpopulations during cyst initiation and progression, as we have done in this study. It is also important to address how *Pkd1* mutant collecting duct cells affect the biology and function of neighboring, genetically normal cells before cyst initiation and during cyst progression, and to systematically investigate gene expression profiles in these cells, which include macrophages, NK/T cells, and fibroblasts in the cyst microenvironment. Ultimately, the identification of genes that are specifically expressed in each cell type will provide a cell-level understanding of the physiology and pathophysiology of PKD, and will provide for the creation of a publicly accessible online resource that will benefit the PKD research community.

## Data Availability

The authors declare that all data supporting the findings of this study are available within the article, the Supplementary Data, and the data repository, or from the corresponding author upon reasonable request.

## Supplementary Materials

The following supporting information can be downloaded at: https://www.mdpi.com/article/10.3390/cells12010045/s1. Figure S1. Ambient RNA and doublets were removed by SouxP and DoubletFinder programs; Figure S2. The list of differentially expressed genes in collecting duct principal (CD-PC) cells at day 14; Figure S3. tSNE plots showing gene expression patterns in proximal tubule, CD principal cells, CD intercalated cells, macrophages, fibroblasts and immune cells; Figure S4. tSNE plots showing gene expression patterns in Loop of Henle, neutrophil and podocytes cells; Figure S5. Cell diversity of collecting duct cells during kidney development and cyst development; Figure S6. Heatmap showing the top DEGs from collecting duct cells at day 7, 14 and 21 Pkd1 heterozygous and homozygous knockout kidneys; Figure S7. Communications mediated by ligand-receptor pairs in day 14 Pkd1 homozygous kidneys; Figure S8. Cell-to-cell communications in collecting duct cell subsets in Pkd1 heterozygous and homozygous knockout kidneys across three stages as analyzed by CellChat program; Table S1. The list of differentially expressed genes in collecting duct principal (CD-PC) cells at day 7; Table S2. The list of differentially expressed genes in collecting duct principal (CD-PC) cells at day 14; Table S3. The list of differentially expressed genes in collecting duct principal (CD-PC) cells at day 21; Table S4. The list of differentially expressed genes in fibroblast cells at day 7; Table S5. The list of differentially expressed genes in fibroblast cells at day 14; Table S6. The list of differentially expressed genes in fibroblast cells at day 21; Table S7. The list of differentially expressed genes in macrophages at day 7; Table S8. The list of differentially expressed genes in macrophages at day 14; Table S9. The list of differentially expressed genes in macrophages at day 21; Table S10. The list of differentially expressed genes in NK and T cells at day 7; Table S11. The list of differentially expressed genes in NK and T cells at day 14; Table S12. The list of differentially expressed genes in NK and T cells at day 21.
