# Supplementary material for: Single-Cell and CellChat Resolution Identifies Collecting Duct Cell Subsets and Their Communications with Adjacent Cells in PKD Kidneys
Source: Cells. 2022 Dec 22;12(1):45. doi: 10.3390/cells12010045 (PMC9818381; doi:10.3390/cells12010045)
Supplement: Supplementary file 1 [file cells-12-00045-s001.zip › Supplemental figure-12-01-2022.pdf]

Supplemental figures and legends

Supplementary Figure S1

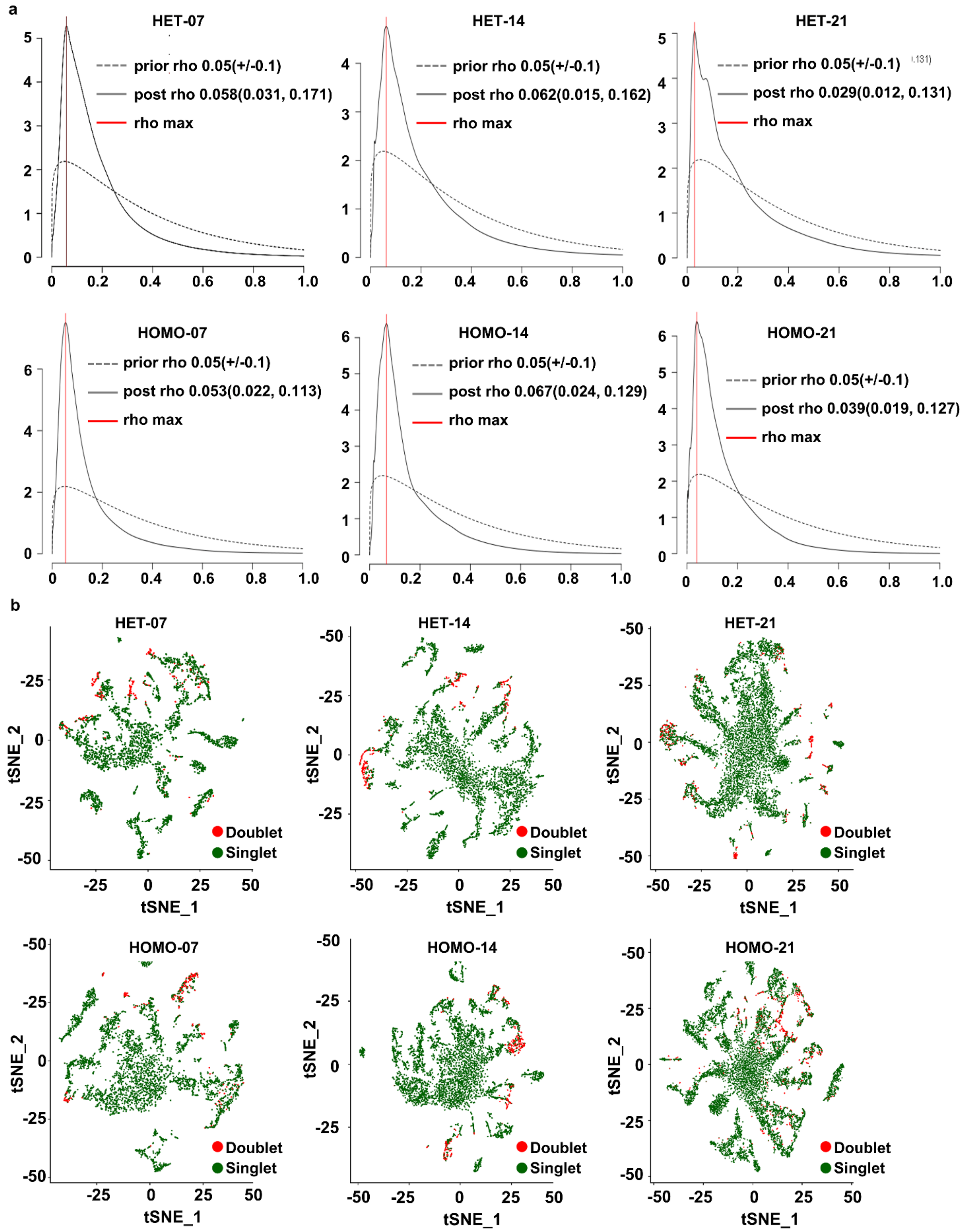

Supplemental Figure S1. Ambient RNA and doublets were removed by SouxP and DoubletFinder programs. (**a**) Representative graphs showed estimated contamination of ambient RNA calculated by SouxP, and expression matrix was filtered with removal of ambient RNA. (**b**) tSNE plots showing doublets determined by DoubletFinder program, and singlets from each group were subset from here for following analysis.

Supplementary Figure S2

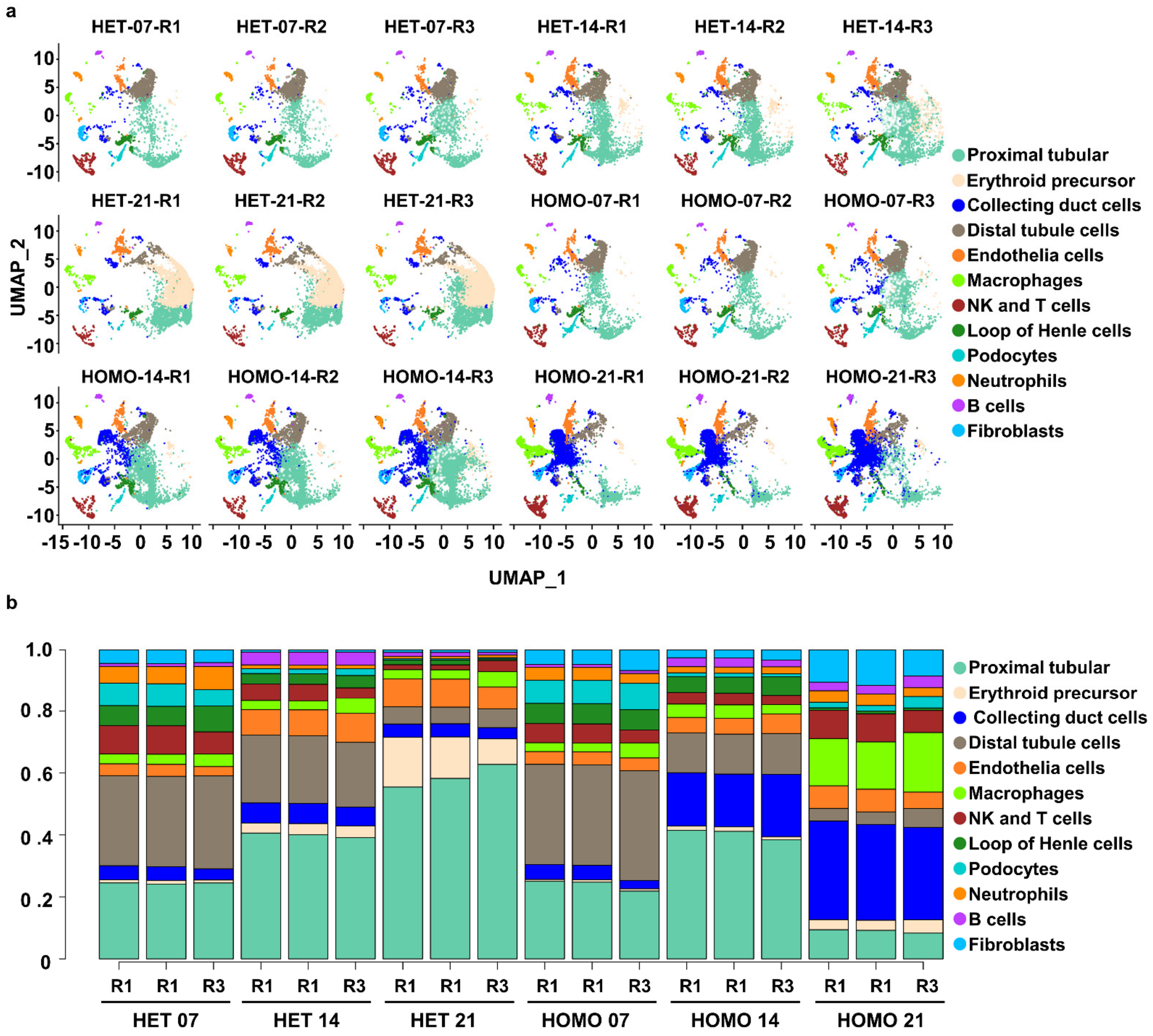

Supplemental Figure S2. The list of differentially expressed genes in collecting duct principal (CD-PC) cells at day 14. Percent cells 1 and 2 represent the percentages of cells expressing the specific gene in *Pkd1* HOMO and HET kidneys, respectively. P-values and average natural log expression differences were calculated using the Seurat package as described in Materials and Methods.

Supplementary Figure S3

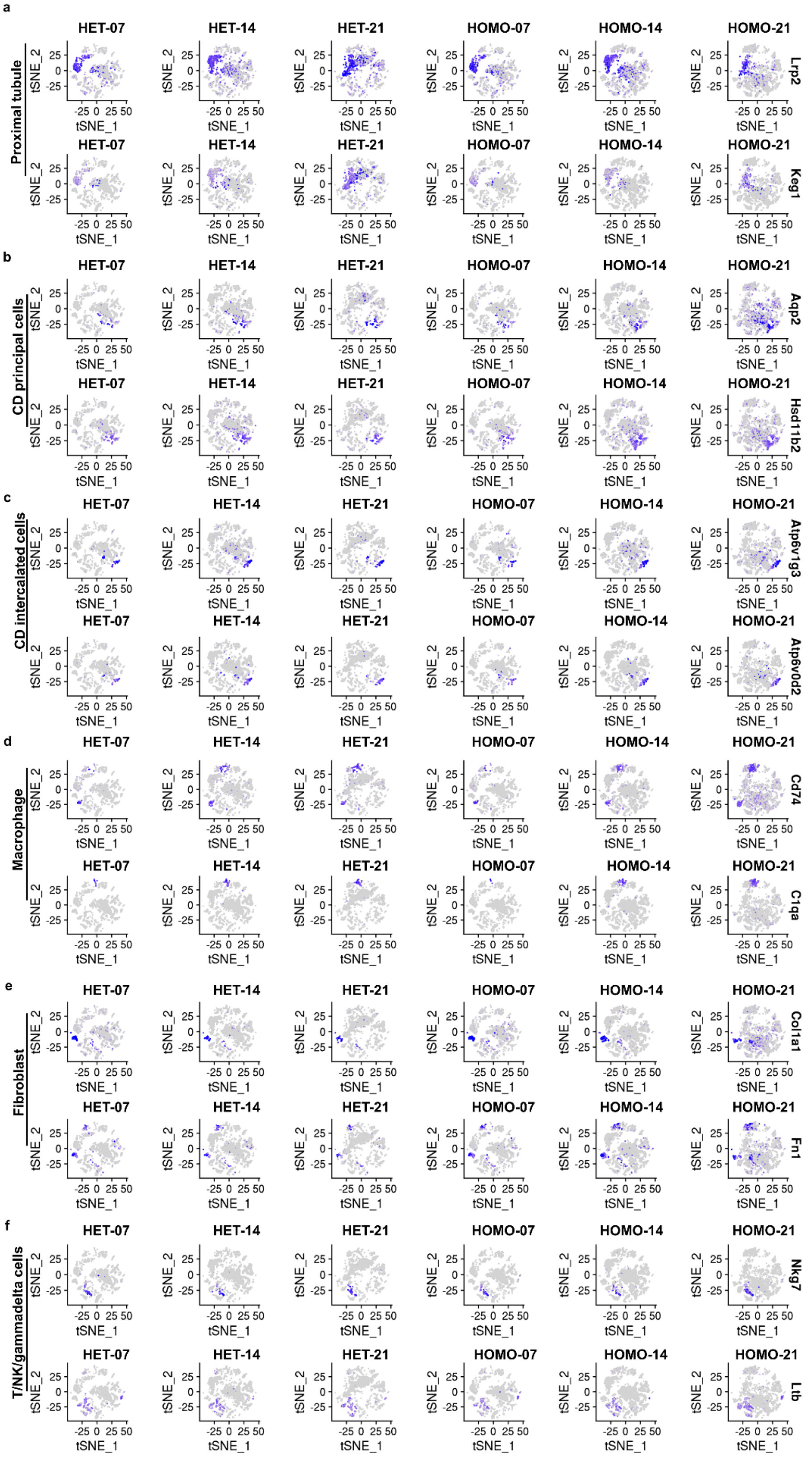

Supplemental Figure S3. tSNE plots showing gene expression patterns in proximal tubule, CD principal cells, CD intercalated cells, macrophages, fibroblasts and immune cells. (**a**) tSNE plots showing gene

expression levels of cell type markers of proximal tubule cells, including Lrp2 and Keg1. (**b**) tSNE plots showing gene expression levels of cell type markers of collecting duct principal cells, including Aqp2 and Hsd11b2. (**c**) tSNE plots showing gene expression levels of cell type markers of collecting duct intercalated cells, including Atp6v1g3 and Atp6v0d2. (**d**) tSNE plots showing gene expression levels of cell type markers of macrophages, including CD74 and C1qa. (**e**) tSNE plots showing gene expression levels of cell type markers of fibroblasts, including Col1a1 and Fn1. (**f**) tSNE plots showing gene expression levels of cell type markers (Nkg7 and Ltb) of immune cells, including NK/T cells.

Supplementary Figure S4

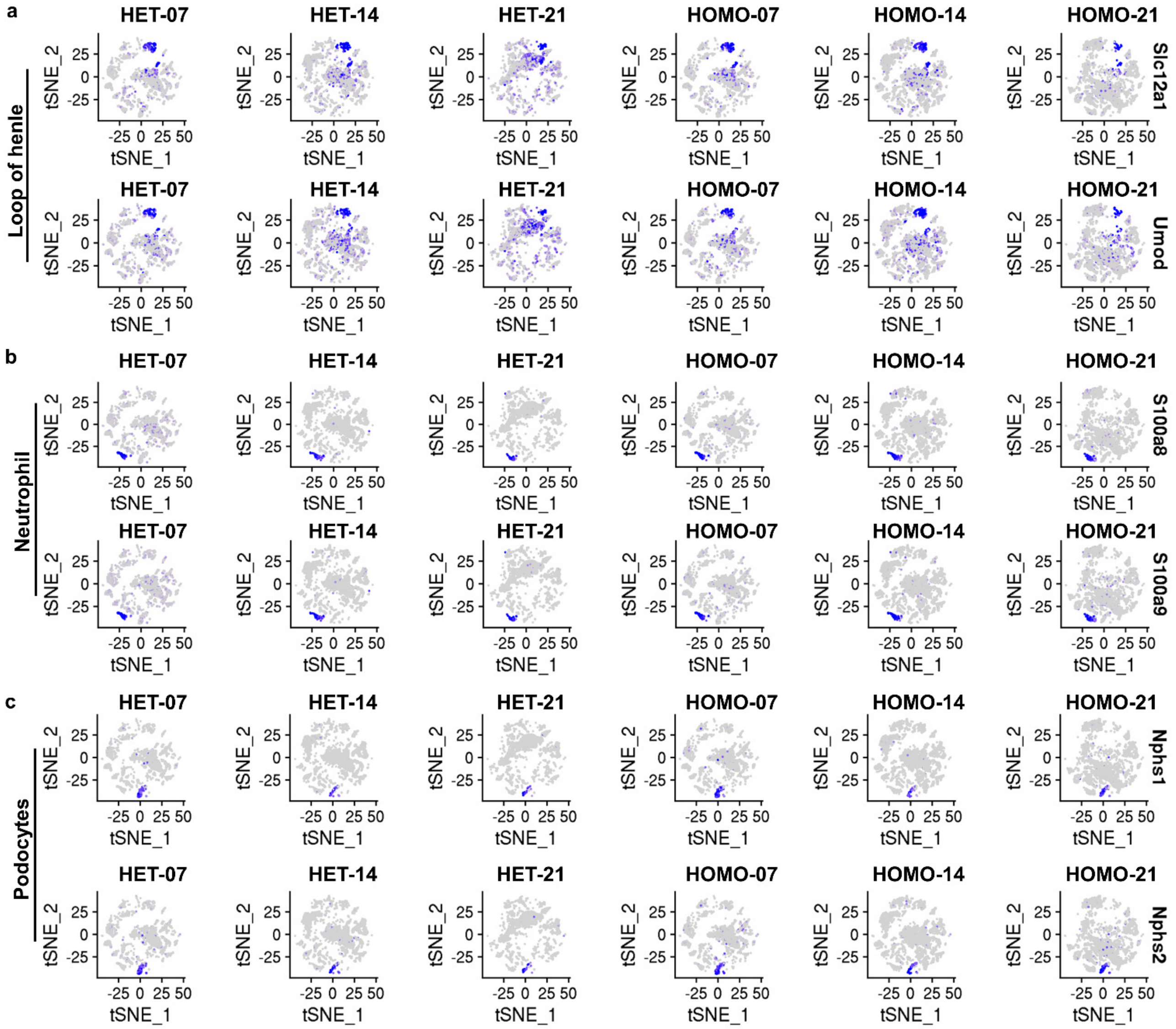

Supplemental Figure S4. tSNE plots showing gene expression patterns in Loop of Henle, neutrophil and podocytes cells. (**a**) tSNE plots showing gene expression levels of cell type markers of Loop of Henle (LOH), including Slc12a1 and Umod. (**b**) tSNE plots showing gene expression levels of cell type markers of neutrophils, including S100a8 and S100a9. (**c**) tSNE plots showing gene expression levels of cell type markers of podocytes, including Nphs1 and Nphs2.

Supplementary Figure S5

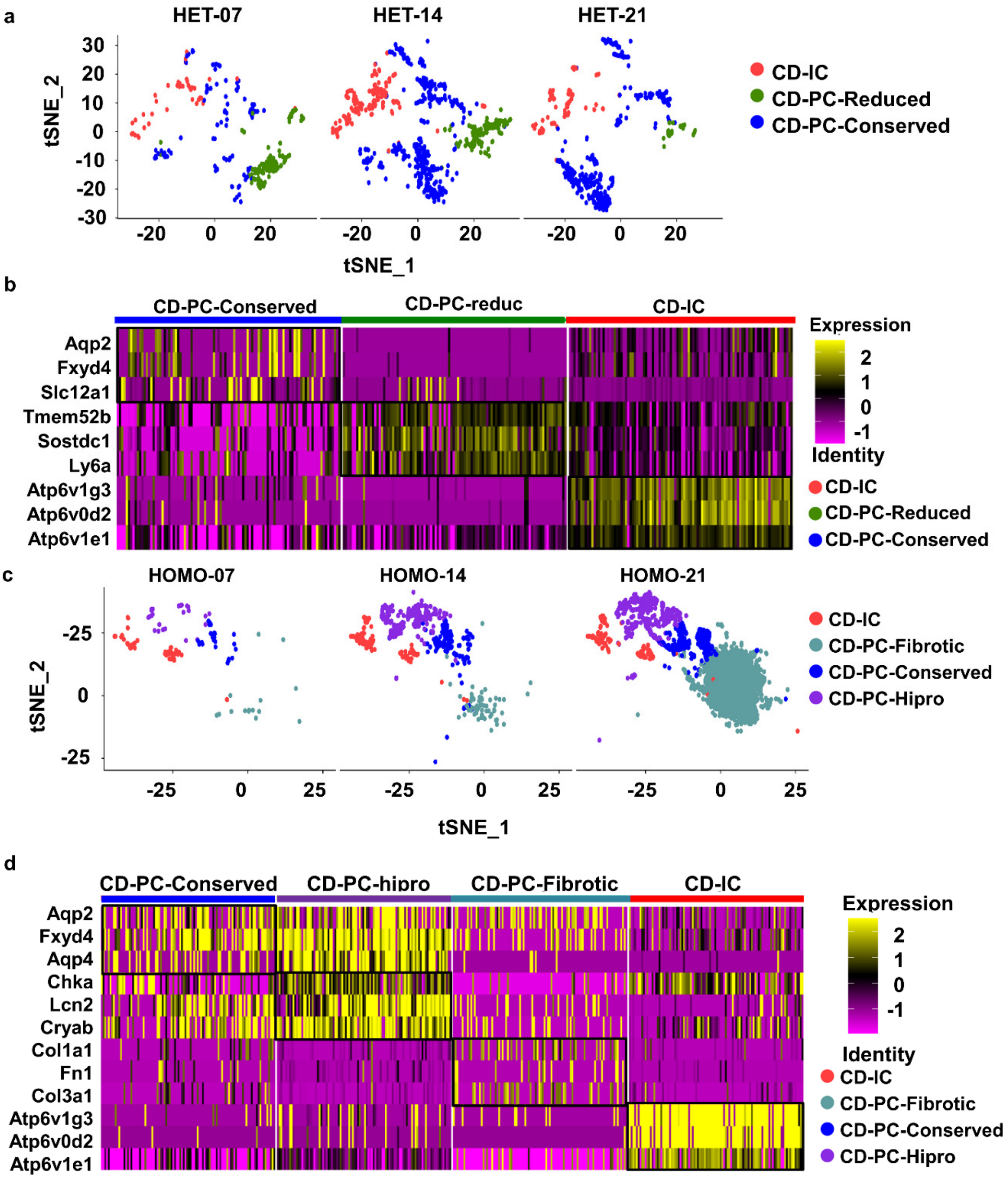

Supplemental Figure S5. Cell diversity of collecting duct cells during kidney development and cyst development. (**a** and **b**) Identification and characterization of collecting duct cell subtypes from day 7, 14 and 21 *Pkd1* HET kidneys. (**a**) tSNE plots showing sub-clustering of collecting duct cells. (**b**) Heatmap shows specific markers for each subtype. Each column represents a cell, and each row represents a gene. (**c** and **d**) Identification and characterization of collecting duct cell subtypes from day 7, 14 and 21 *Pkd1* HOMO kidneys. (**c**) tSNE plots showing sub-clustering of collecting duct cells. (**d**) Heatmap shows specific markers for each subtype of collecting ducts. Each column represents a cell, and each row represents a gene.

Supplementary Figure S6

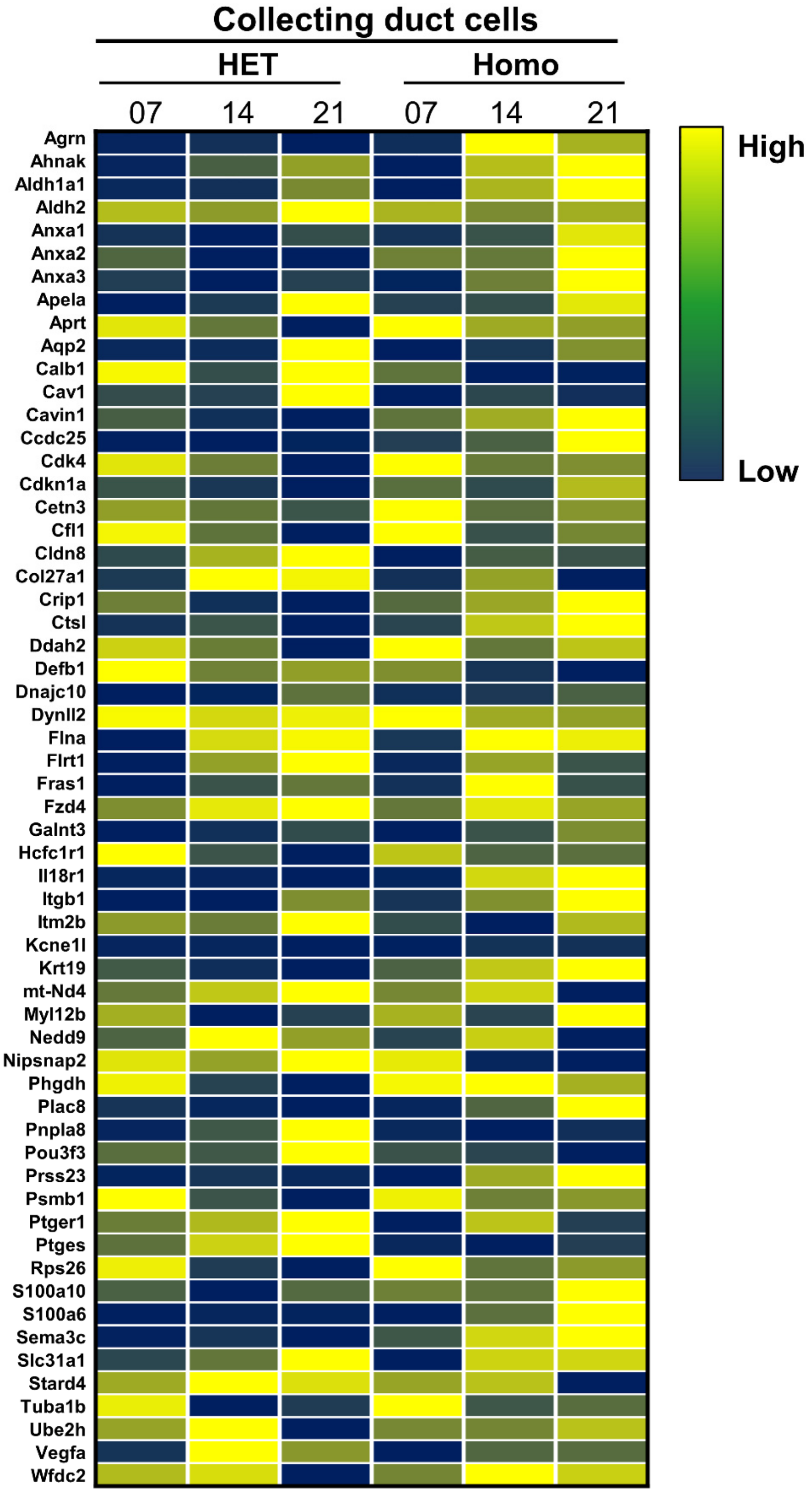

Supplemental Figure S6. Heatmap showing the top DEGs from collecting duct cells at day 7, 14 and 21 *Pkd1* heterozygous and homozygous knockout kidneys.

Supplementary Figure S7

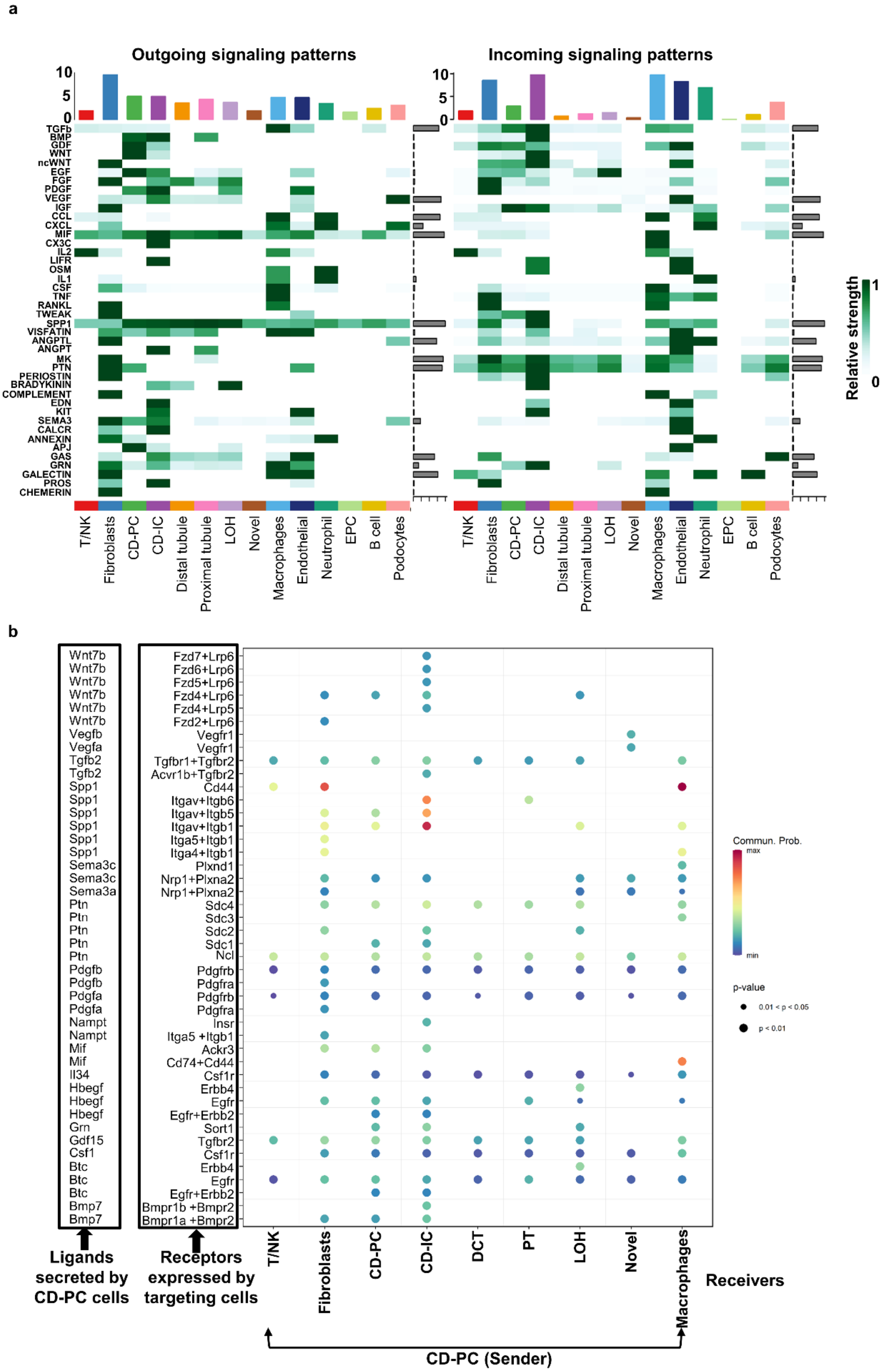

Supplemental Figure S7. Communications mediated by ligand-receptor pairs in day 14 *Pkd1* homozygous kidneys. (**a**) Heatmaps showing identified signals (ligands) contributing most to outgoing or incoming signaling of certain cell type. **(b)** Bubble plot showing all the significant interactions (L-R pairs)

mediated by the signals (ligands) sending from CD-PC cells and receiving by their receptors on other cell types. Color bar represents the communication probabilities mediated by ligand-receptor pairs from CD-PC cells to other cell types.

Supplementary Figure S8

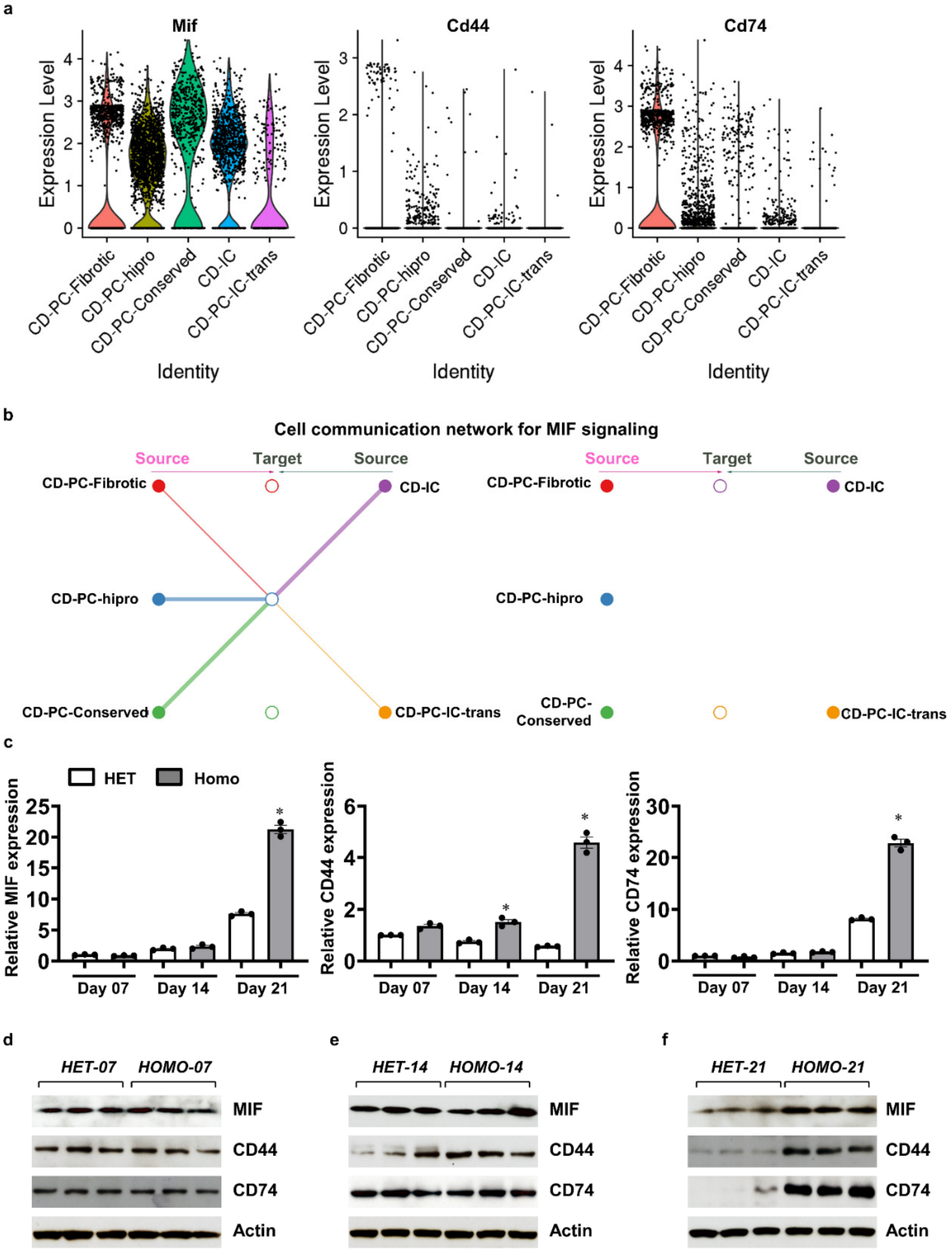

Supplemental Figure S8. Cell-to-cell communications in collecting duct cell subsets in Pkd1 heterozygous and homozygous knockout kidneys across three stages as analyzed by CellChat program. (**a**) Violin plots showing MIF and its receptors CD74 and CD44 dynamic expression in five cell

subtypes of collecting duct cells during PKD progression. (**b**) Hierarchical plots show the inferred intercellular communication network for MIF signaling. Solid and open circles represent source and target cell types, respectively. Circle sizes are proportional to the number of cells in each cell subtype. Edge colors of middle circles are consistent with the signaling source. (**c**) qRT-PCR analysis of relative MIF, CD74 and CD44 mRNA expression in day 7, 14 and 21 *Pkd1* HET and *Pkd1* HOMO kidneys. * represents the comparison between *Pkd1* HOMO kidneys and age-matched *Pkd1* HET kidneys ($p < 0.05$), as calculated by a one-way ANOVA test. (**d-f**) Western blot analysis of MIF, CD74 and CD44 protein in day 7, 14 and 21 *Pkd1* HET and *Pkd1* HOMO kidneys.

Supplemental Tables.

Supplemental Table S1. The list of differentially expressed genes in collecting duct principal (CD-PC) cells at day 7. Percent cells 1 and 2 represent the percentages of cells expressing the specific gene in *Pkd1* HOMO and HET kidneys, respectively. P-values and average natural log expression differences were calculated using the Seurat package as described in Materials and Methods.

Supplemental Table S2. The list of differentially expressed genes in collecting duct principal (CD-PC) cells at day 14. Percent cells 1 and 2 represent the percentages of cells expressing the specific gene in *Pkd1* HOMO and HET kidneys, respectively. P-values and average natural log expression differences were calculated using the Seurat package as described in Materials and Methods.

Supplemental Table S3. The list of differentially expressed genes in collecting duct principal (CD-PC) cells at day 21. Percent cells 1 and 2 represent the percentages of cells expressing the specific gene in *Pkd1* HOMO and HET kidneys, respectively. P-values and average natural log expression differences were calculated using the Seurat package as described in Materials and Methods.

Supplemental Table S4. The list of differentially expressed genes in fibroblast cells at day 7. Percent cells 1 and 2 represent the percentages of cells expressing the specific gene in *Pkd1* HOMO and HET kidneys, respectively. P-values and average natural log expression differences were calculated using the Seurat package as described in Materials and Methods.

Supplemental Table S5. The list of differentially expressed genes in fibroblast cells at day 14. Percent cells 1 and 2 represent the percentages of cells expressing the specific gene in *Pkd1* HOMO and HET kidneys, respectively. P-values and average natural log expression differences were calculated using the Seurat package as described in Materials and Methods.

Supplemental Table S6. The list of differentially expressed genes in fibroblast cells at day 21. Percent cells 1 and 2 represent the percentages of cells expressing the specific gene in *Pkd1* HOMO and HET kidneys, respectively. P-values and average natural log expression differences were calculated using the Seurat package as described in Materials and Methods.

Supplemental Table S7. The list of differentially expressed genes in macrophages at day 7. Percent cells 1 and 2 represent the percentages of cells expressing the specific gene in *Pkd1* HOMO and HET kidneys, respectively. P-values and average natural log expression differences were calculated using the Seurat package as described in Materials and Methods.

Supplemental Table S8. The list of differentially expressed genes in macrophages at day 14. Percent cells 1 and 2 represent the percentages of cells expressing the specific gene in *Pkd1* HOMO and HET kidneys,

respectively. P-values and average natural log expression differences were calculated using the Seurat package as described in Materials and Methods.

Supplemental Table S9. The list of differentially expressed genes in macrophages at day 21. Percent cells 1 and 2 represent the percentages of cells expressing the specific gene in *Pkd1* HOMO and HET kidneys, respectively. P-values and average natural log expression differences were calculated using the Seurat package as described in Materials and Methods.

Supplemental Table S10. The list of differentially expressed genes in NK and T cells at day 7. Percent cells 1 and 2 represent the percentages of cells expressing the specific gene in *Pkd1* HOMO and HET kidneys, respectively. P-values and average natural log expression differences were calculated using the Seurat package as described in Materials and Methods.

Supplemental Table S11. The list of differentially expressed genes in NK and T cells at day 14. Percent cells 1 and 2 represent the percentages of cells expressing the specific gene in *Pkd1* HOMO and HET kidneys, respectively. P-values and average natural log expression differences were calculated using the Seurat package as described in Materials and Methods.

Supplemental Table S12. The list of differentially expressed genes in NK and T cells at day 21. Percent cells 1 and 2 represent the percentages of cells expressing the specific gene in *Pkd1* HOMO and HET kidneys, respectively. P-values and average natural log expression differences were calculated using the Seurat package as described in Materials and Methods.
