# Supplementary figures and images for: Single-Cell and CellChat Resolution Identifies Collecting Duct Cell Subsets and Their Communications with Adjacent Cells in PKD Kidneys
Source: Cells. 2022 Dec 22;12(1):45. doi: 10.3390/cells12010045 (PMC9818381; doi:10.3390/cells12010045)

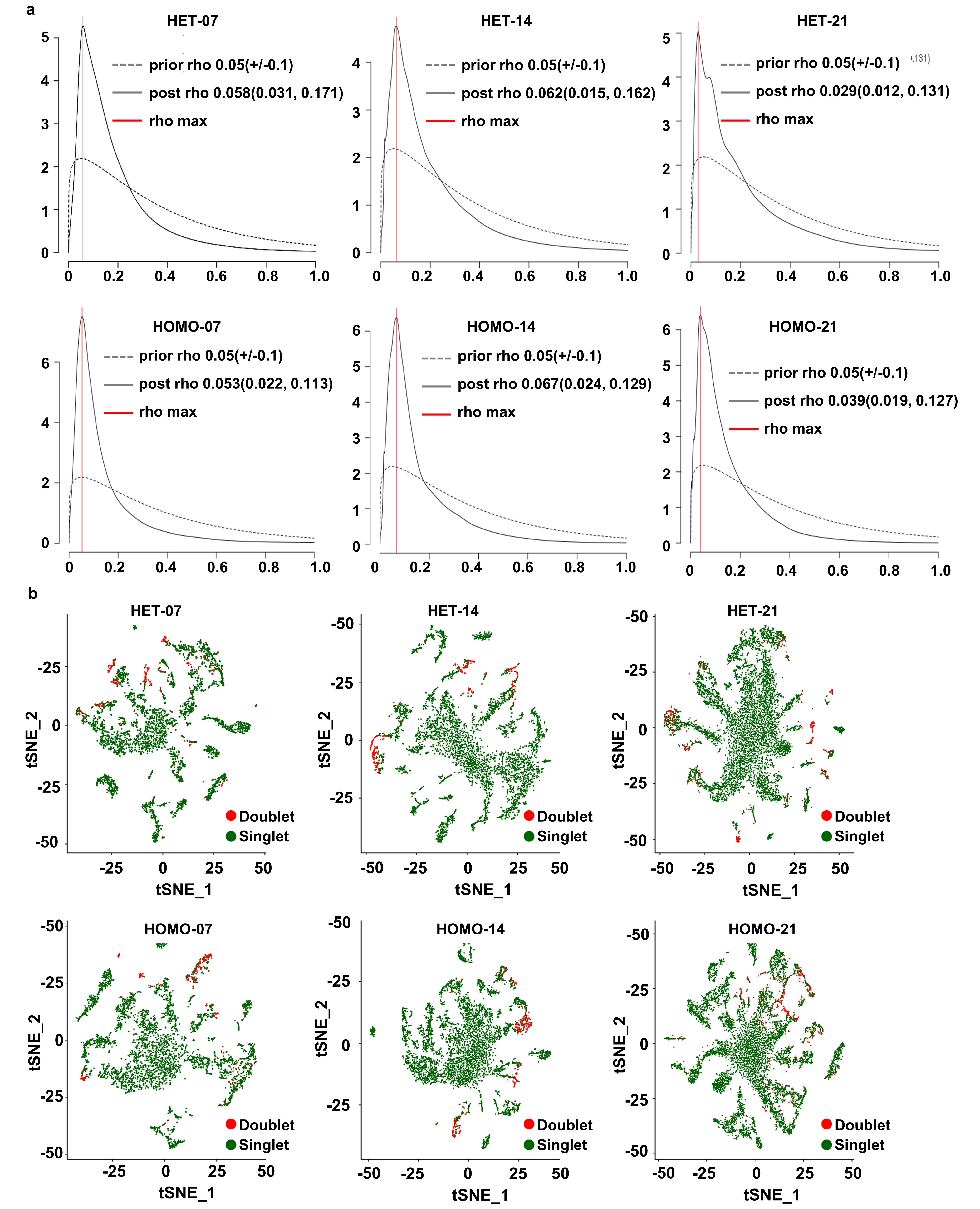

Supplement: Supplementary file 1 [file cells-12-00045-s001.zip › figure S1.tif]

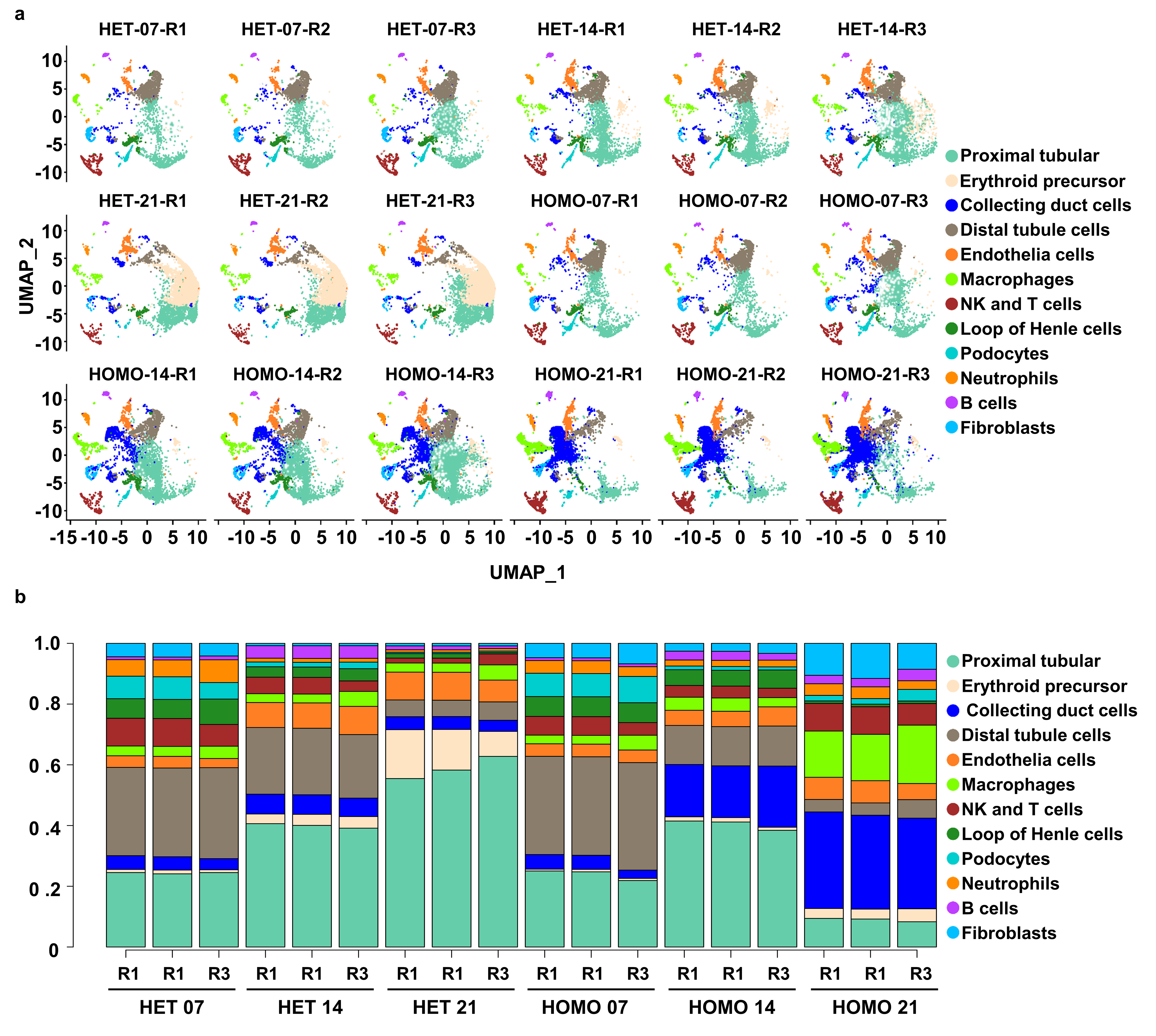

Supplement: Supplementary file 1 [file cells-12-00045-s001.zip › figure S2.tif]

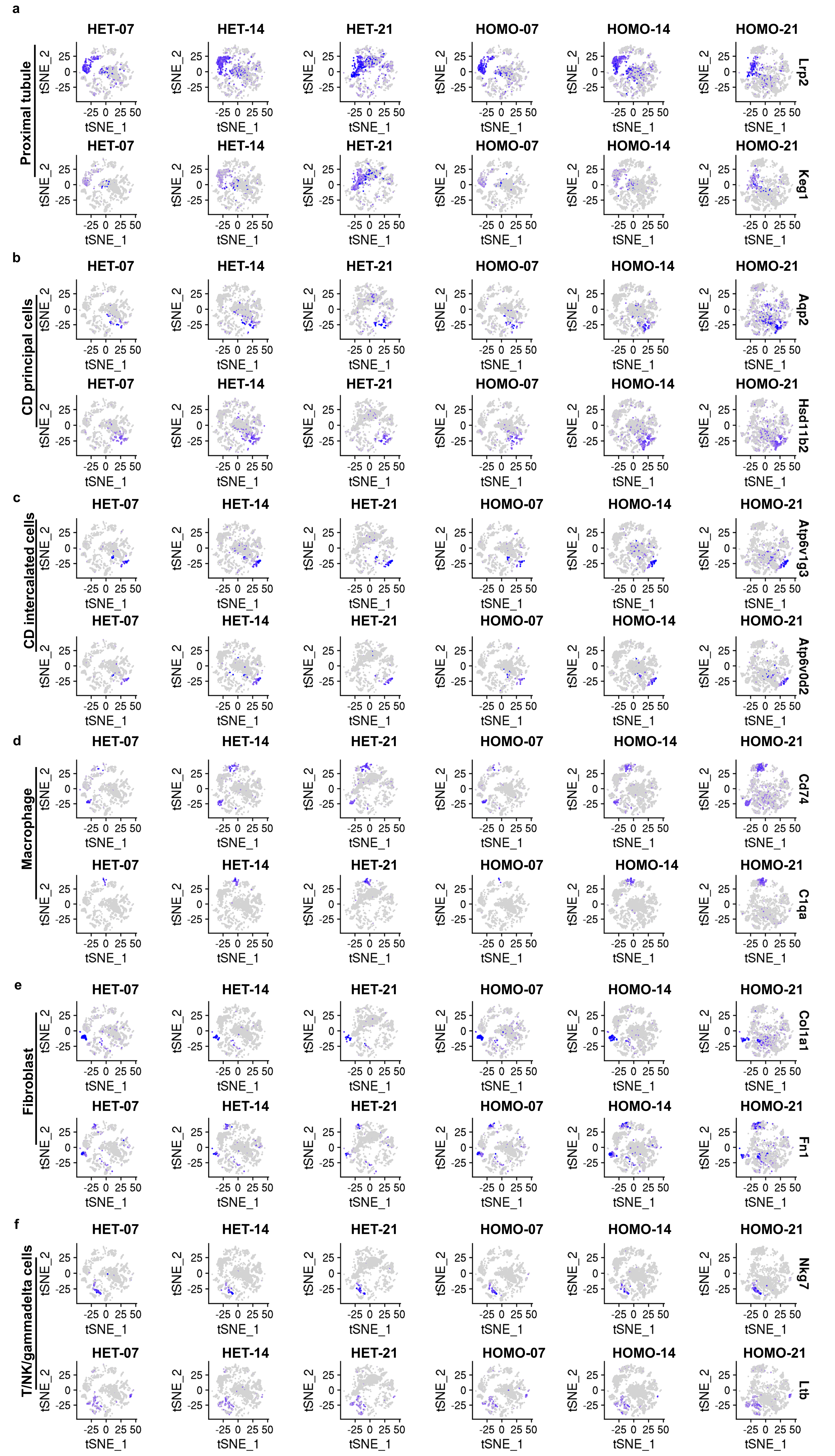

Supplement: Supplementary file 1 [file cells-12-00045-s001.zip › figure S3.tif]

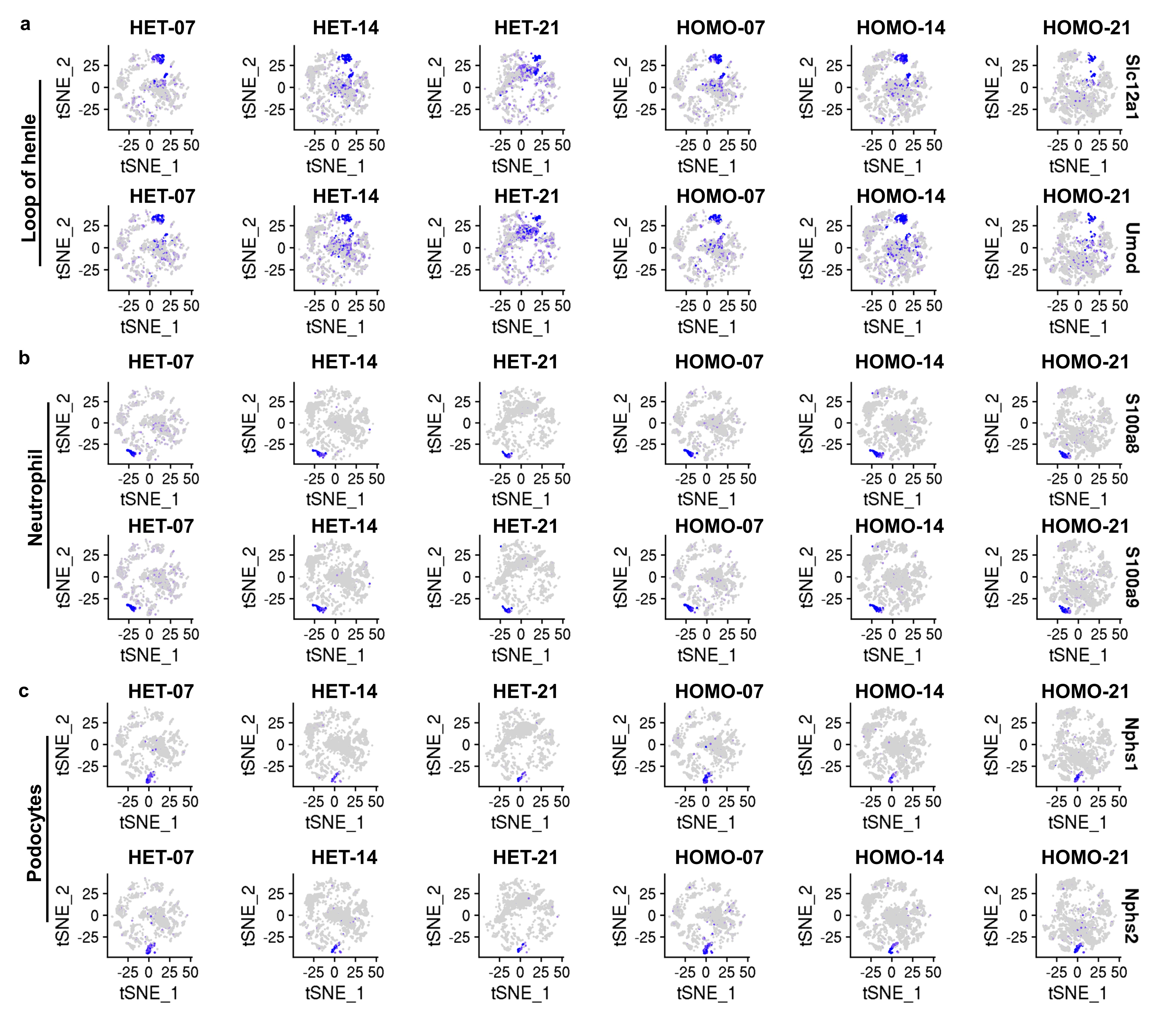

Supplement: Supplementary file 1 [file cells-12-00045-s001.zip › figure S4.tif]

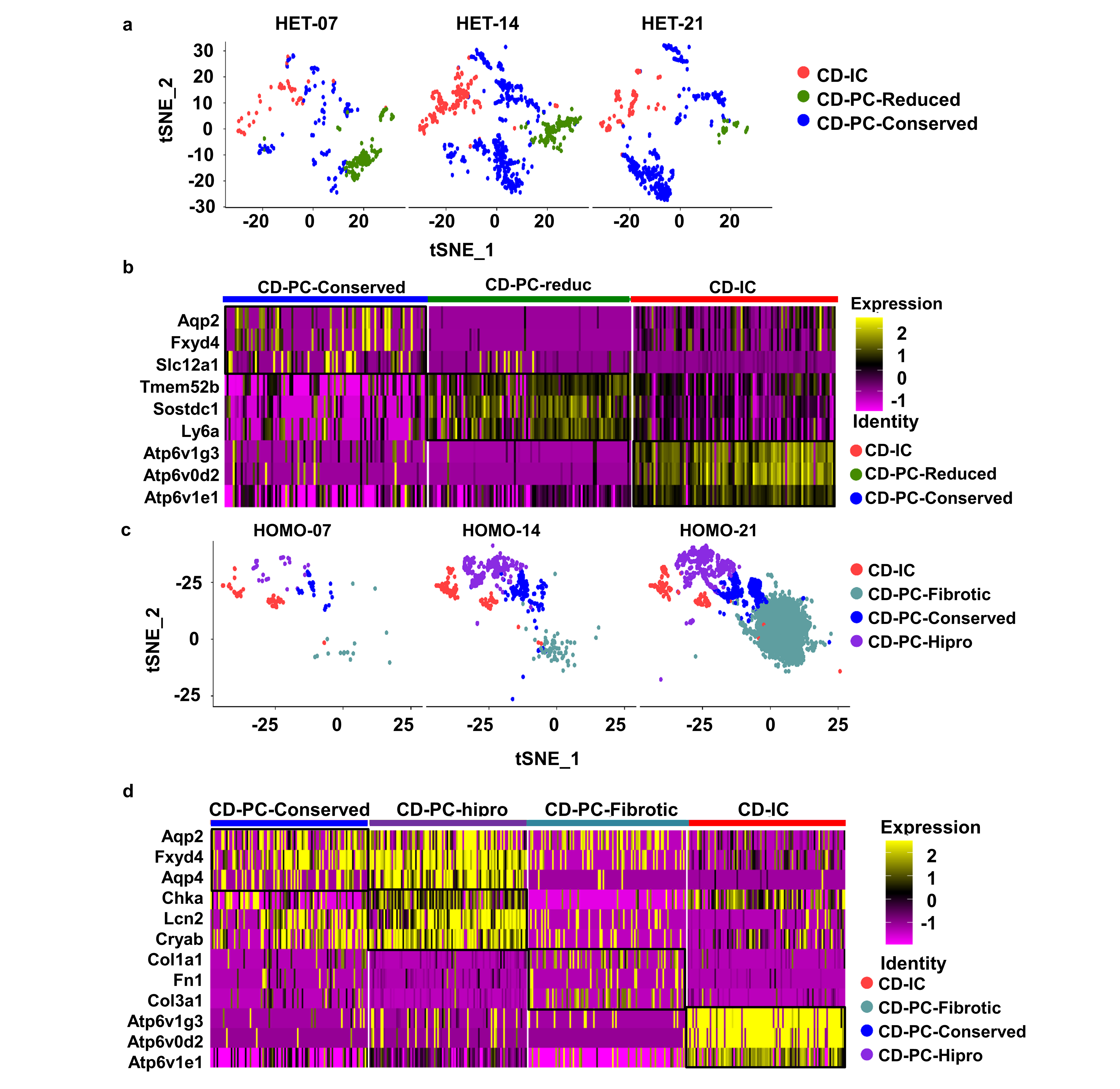

Supplement: Supplementary file 1 [file cells-12-00045-s001.zip › figure S5.tif]

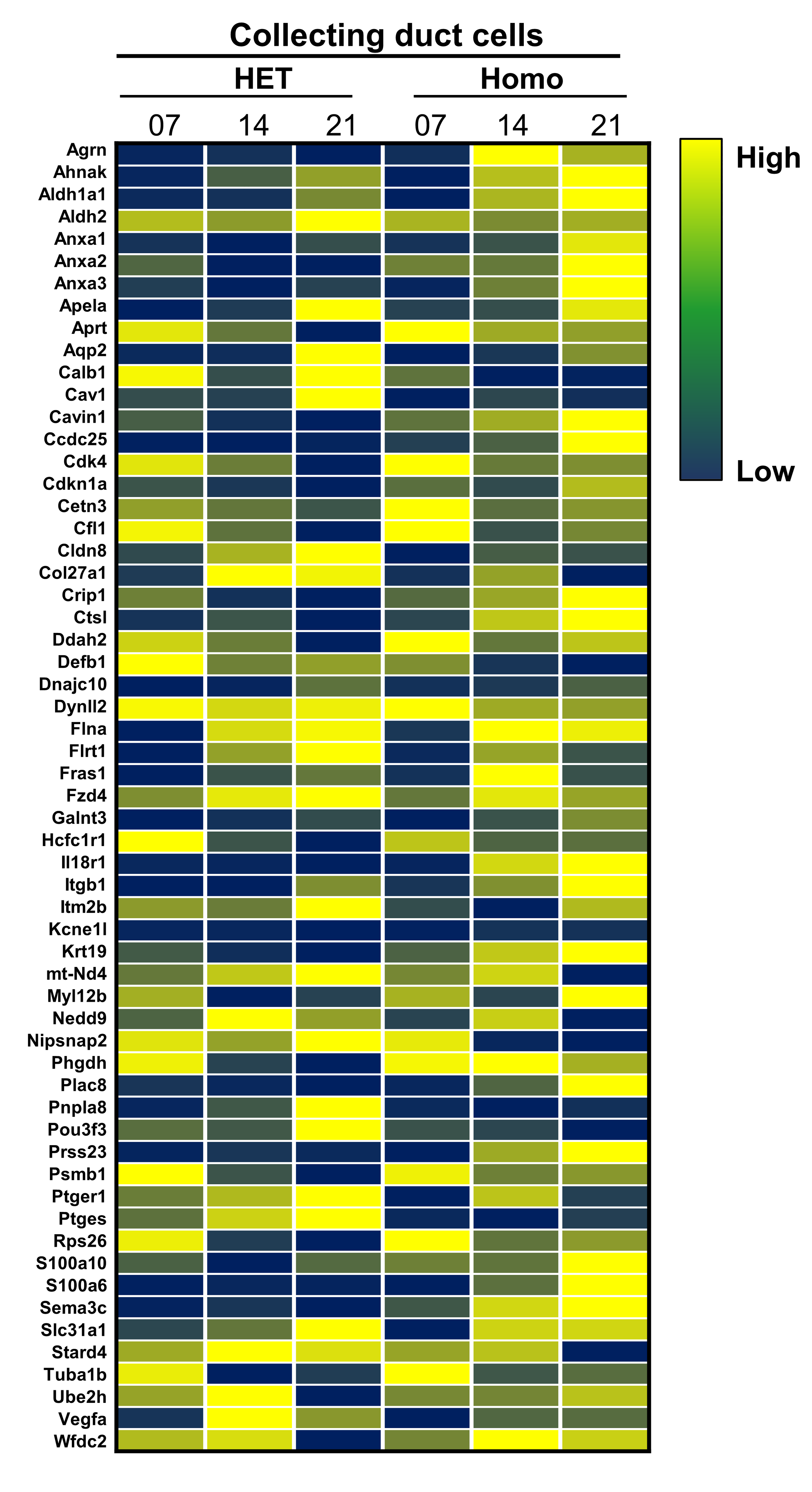

Supplement: Supplementary file 1 [file cells-12-00045-s001.zip › figure S6.tif]

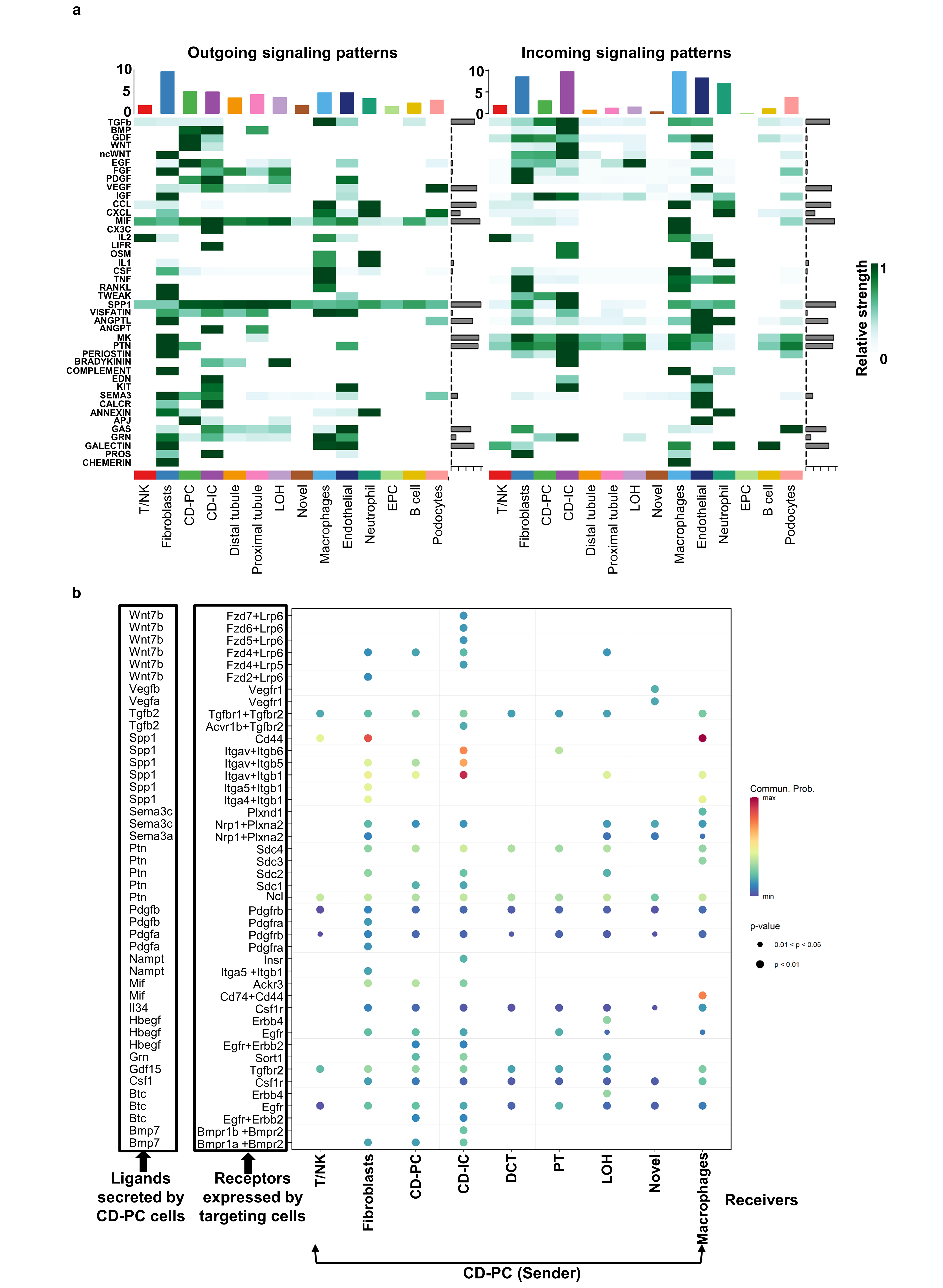

Supplement: Supplementary file 1 [file cells-12-00045-s001.zip › figure S7.tif]

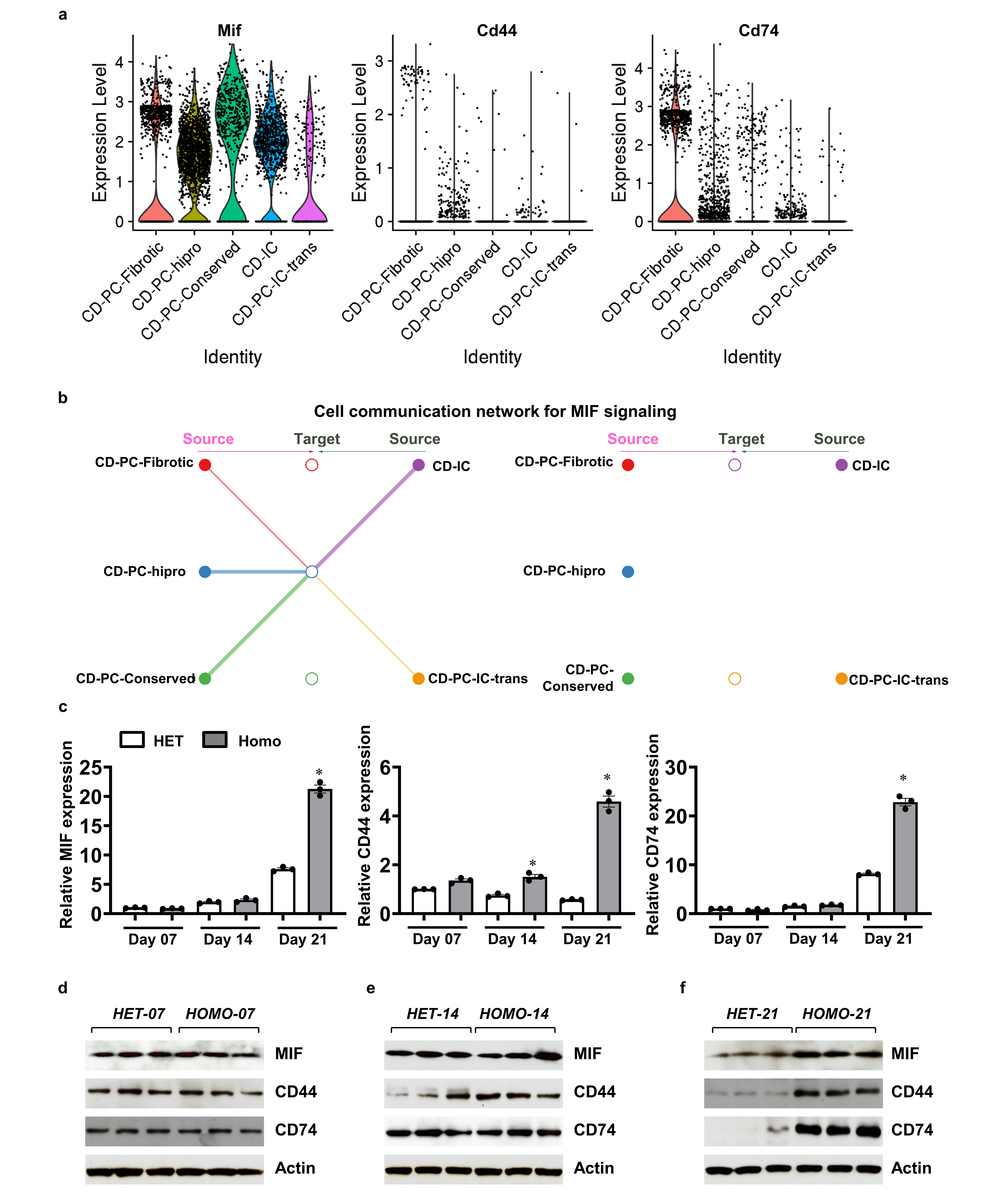

Supplement: Supplementary file 1 [file cells-12-00045-s001.zip › figure S8.tif]
